# Machine Learning-Identified Potential Interaction Between Clazosentan and Nicardipine in Patients with Subarachnoid Hemorrhage

**DOI:** 10.3390/jcm15041383

**Published:** 2026-02-10

**Authors:** Yusuke Inoue, Masahito Katsuki, Toshikazu Hidaka, Junichiro Ochiai, Yuichiro Kawamoto, Daizo Ishii, Katsumi Takizawa, Hirofumi Nakatomi, Masaki Chin, Motohiro Morioka, Hiroki Kurita, Kaima Suzuki, Takatoshi Sorimachi, Koreaki Irie, Ichiro Nakahara, Nobutaka Horie, Fusao Ikawa

**Affiliations:** 1 Department of Neurosurgery, Shimane Prefectural Central Hospital, Izumo 693-8555, Shimane, Japan; yusukeinoue0610@outlook.jp (Y.I.);; 2 Physical Education and Health Center, Nagaoka University of Technology, Nagaoka 940-2137, Niigata, Japan; 3 School of Health and Human Performance, Dublin City University, D09 YT18 Dublin, Ireland; 4 Insight Research Ireland Centre for Data Analytics, Dublin City University, D09 YT18 Dublin, Ireland; 5 Department of Neurosurgery, Graduate School of Biomedical and Health Sciences, Hiroshima University, Hiroshima 734-0037, Hiroshima, Japan; 6 Department of Neurosurgery, Japanese Red Cross Asahikawa Hospital, Asahikawa 070-0061, Hokkaido, Japan; 7 Department of Neurosurgery, Kyorin University, Mitaka 181-8611, Tokyo, Japan; 8 Department of Neurosurgery, International University of Health and Welfare, Nasushiobara 324-8501, Tochigi, Japan; 9 Department of Neurosurgery, Kurashiki Central Hospital, Kurashiki 710-0052, Okayama, Japan; 10 Department of Neurosurgery, Kurume University School of Medicine, Kurume 830-0011, Fukuoka, Japan; 11 Department of Cerebrovascular Surgery, Saitama Medical University International Medical Center, Hidaka 350-1298, Saitama, Japan; 12 Medical Corporation Chiseikai, Suzuki Neurosurgery, Neuroscience Center, Kawagoe 350-1175, Saitama, Japan; 13 Department of Neurosurgery, Tokai University, Isehara 259-1193, Kanagawa, Japan; 14 Department of Neurosurgery, Japan Red Cross Medical Center, Shibuya-ku 150-8935, Tokyo, Japan; 15 Department of Neurosurgery, Fujita Health University Bantane Hospital, Nagoya 454-8509, Aichi, Japan

**Keywords:** cerebral vasospasm (VS), clazosentan, interactions, machine learning, nicardipine, subarachnoid hemorrhage (SAH)

## Abstract

**Background/Objectives**: Subarachnoid hemorrhage (SAH) is frequently complicated by cerebral vasospasm (VS). Clazosentan has reduced VS in Japanese studies but shown inconsistent efficacy in Western trials. We hypothesized that clinical and pharmacologic interactions may influence its effectiveness. **Methods**: We analyzed the multicenter “Database of Cohort Study for Outcome of SAH In Japan” (DCI Japan) registry, prospectively collected from 2020 to 2023, to assess associations between clazosentan use, VS prevention, functional outcomes, and potential interactions in adults with aneurysmal SAH (aSAH) treated by surgical clipping or endovascular coiling within 4 days of onset. Outcomes included angiographic VS (AVS), symptomatic VS (SVS), cerebral infarction, and modified Rankin Scale (mRS) scores at discharge and at 6 months. Predictors and interactions were first screened using univariable analysis and Light Gradient Boosting Machine, then evaluated via multivariable logistic regression. **Results**: Among 544 patients (mean age 65.2 ± 14.2 years; 71.5% female), 34.0% received clazosentan. AVS, SVS, and cerebral infarction occurred in 20.6%, 16.0%, and 22.4%, respectively. Poor outcomes (mRS 3–6) were observed in 48.8% at discharge and 33.7% (137/406) at 6 months. Clazosentan use was associated with reduced odds of AVS (OR 0.27, 95% CI [0.11–0.69]), SVS (OR 0.15 [0.04–0.64]), and poor 6-month outcome (OR 0.08 [0.01–0.68]). A potential interaction with nicardipine was linked to higher odds of AVS (OR 1.85 [1.43–2.65]). **Conclusions**: Clazosentan was associated with reduced VS and improved 6-month outcomes after aSAH, although concomitant nicardipine may attenuate its prophylactic effectiveness against AVS.

## 1. Introduction

Subarachnoid hemorrhage (SAH) is a severe neurological disorder associated with high morbidity and mortality [1,2,3,4]. Cerebral vasospasm (VS), a major complication of SAH, contributes to delayed cerebral ischemia (DCI) and poor clinical outcomes [5]. VS develops in 30–70% of SAH cases and results in stroke or death in 15–20% [6]. Although the pathogenesis of DCI is multifactorial [7], prevention of VS remains an important therapeutic goal. Current pharmacologic strategies include nimodipine, a calcium channel blocker (CCB) widely used in Western countries [8,9], and fasudil hydrochloride (fasudil), a Rho kinase inhibitor commonly prescribed in Japan [10,11].

Clazosentan, a selective endothelin A (ETA) receptor antagonist, inhibits endothelin-1-mediated vasoconstriction. While the CONSCIOUS trials demonstrated its ability to reduce VS, it showed limited impact on functional outcome and mortality [12,13,14]. Similarly, the REACT trial [15] did not demonstrate a significant reduction in DCI. Recent meta-analyses have confirmed that although clazosentan lowers the incidence of VS, it does not significantly improve clinical outcomes [16,17,18]. In contrast, a randomized controlled trial (RCT) conducted in Japan [19] reported that clazosentan reduced VS and improved 12-week clinical outcomes, which were further supported by other retrospective studies [20,21].

Given these discrepant RCT results across countries, we hypothesized that clinical and pharmacologic contexts, including potential drug–drug interactions, may influence the effectiveness of clazosentan. Accordingly, this multicenter observational study examined clazosentan’s effect on VS prevention and functional outcomes and explored potential interactions influencing clazosentan’s effect.

## 2. Materials and Methods

### 2.1. Ethical Considerations

This multicenter study was approved by the ethics committees/Institutional Review Boards of all participating institutions. Written informed consent was waived under an institutional opt-out policy. The study adhered to the Declaration of Helsinki and was reported in accordance with STROBE guidelines.

### 2.2. Dataset Information

This study used data from the “Database of Cohort Study for Outcome of SAH In Japan (DCI Japan),” a multicenter registry of ten high-volume cerebrovascular centers in Japan. Details of the DCI-Japan study protocol are provided in Supplementary Method S1. Consecutive cases were enrolled between January 2020 and December 2023. The registry includes patient baseline characteristics, SAH treatment details (e.g., surgery and VS prevention), complications, VS occurrence, and functional outcomes indicated by the modified Rankin Scale (mRS).

### 2.3. Consistent Treatment Protocol of General Management for Aneurysmal SAH

Treatment protocol for aneurysmal SAH (aSAH) in all institutions followed the Japanese Guidelines for the Management of Stroke 2021 [22]. Preoperative management included nicardipine to maintain normovolemia and systolic blood pressure <130 mmHg. Surgical indications followed the Japanese Guidelines for the Management of Stroke 2021: patients with World Federation of Neurosurgical Societies (WFNS) grades I–III were eligible for aneurysm treatment, whereas those with grades IV–V were generally excluded unless they were young or middle-aged, or had a significant hematoma or hydrocephalus. Definitive treatment (surgical clipping or endovascular coiling) was performed within 4 days of onset by certified neurosurgeons following a thorough discussion. Postoperative care included prevention of VS, rehabilitation, and management of complications. SVS was treated with intra-arterial fasudil or percutaneous transluminal angioplasty as needed.

### 2.4. Inclusion and Exclusion Criteria

**Figure 1:**
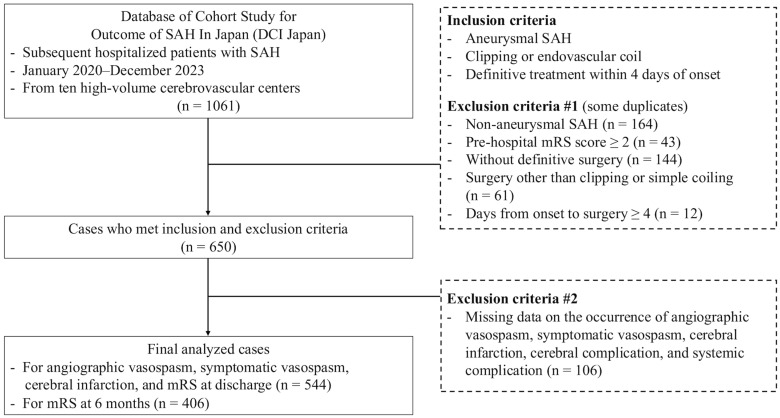
A flow diagram showing patient selection and exclusion criteria for this study. Of 1061 patients enrolled in the DCI Japan registry, 544 with aneurysmal subarachnoid hemorrhage (aSAH) who met the inclusion criteria were included in the analysis. Abbreviations: mRS, modified Rankin Scale.

A diagram of patient selection is shown in Figure 1. This study included patients with aSAH who underwent surgical clipping or endovascular coil embolization for a ruptured aneurysm within 4 days of symptom onset. Exclusion criteria were as follows: diagnostic imaging failed to detect a ruptured cerebral aneurysm; the presence of a non-saccular aneurysm (e.g., dissecting aneurysm, fusiform, infectious, or traumatic pseudoaneurysm); a prehospital mRS score not available or ≥2; cases without definitive treatment, such as only conservation with cerebrospinal fluid drainage; treatment other than simple surgical clipping or simple endovascular coiling including wrapping, trap and bypass surgery, proximal parent artery occlusion, decompression or hematoma removal, or endovascular coiling with stent; complex treatment or treatment associated with failure of one technique and success of the other; missing data on the presence of angiographic VS (AVS), symptomatic VS (SVS), or cerebral infarction; missing or inaccurate date of onset or treatment date, or ≥4 days from onset to surgery; the absence of treatment with either clazosentan or fasudil during the VS period; or deficiencies in imaging data.

### 2.5. Definition of VS

Cerebral VS was assessed using computed tomography (CT) angiography or digital subtraction angiography according to local protocols, typically immediately after surgery, on postoperative days 1, 7, and 14, and when new neurological deficits emerged. The classification into AVS, SVS, and cerebral infarction followed the conventional model of vasoconstriction leading to ischemia [23].

AVS was defined as ≥50% narrowing of any cerebral artery on angiography compared with the preoperative baseline, excluding changes attributable to atherosclerosis, catheter-induced spasm, or vessel hypoplasia [24,25]. Diagnosis was based solely on imaging [26]. SVS was defined as neurological deficits associated with AVS; the appearance of focal neurological signs, or a ≥2-point decrease in the Glasgow Coma Scale sustained for at least 1 h, or both, was considered indicative of ischemia attributable to VS after excluding other potential causes of worsening (e.g., hydrocephalus, seizures, metabolic derangement, infection, or oversedation) [26]. This definition was consistent with that of DCI [26].

Cerebral infarction was defined as new infarcts occurring within 6 weeks of SAH on CT, magnetic resonance imaging, or autopsy, which were absent on early postoperative imaging and not attributable to surgery, catheters, or hematoma. Infarcts from non-VS mechanisms (e.g., cardioembolism, atherothrombosis) were also included [26]. Because this registry did not collect the information on infarction mechanism, we were unable to distinguish VS-attributable infarction from other causes in the present analyses.

### 2.6. Management of VS and Variable Definition of Prophylaxis

Before the approval and availability of clazosentan in Japan (April 2022), VS was managed with intravenous fasudil (90 mg/day for 14 days) [26]. After approval, clazosentan (10 mg/h for 14 days) was administered within 24 h of definitive aneurysm repair as prophylaxis in eligible patients, based on clinical availability and physician discretion, and was not used reactively for established VS.

Additional therapies—including fasudil, cilostazol, statins (rosuvastatin, atorvastatin, pitavastatin), and intravenous nicardipine—were administered per institutional prophylaxis protocols [27]. For this study, “prophylaxis during the VS period” was defined as treatment initiated the day after surgery and continued for 14 days, while reactive use after documented AVS or SVS was coded separately. Concomitant prophylaxis could be administered with clazosentan [27]; when used, these agents were typically started during the same postoperative prophylaxis window rather than sequentially after documented AVS/SVS.

Early discontinuation was flagged. “Completion” indicated continuation of the assigned prophylaxis for the planned 14-day course, whereas “early discontinuation” indicated termination before day 14 for any reason, such as adverse events. In this study, we restricted the primary exposure definition to completed prophylaxis courses (completion) and treated early discontinuation as non-exposure.

In Japan, nicardipine is routinely used for preoperative blood pressure reduction in most patients; therefore, preoperative nicardipine use was not used as an exposure variable in this study. Here, “nicardipine use” was defined only as continuous intravenous nicardipine administration during the VS period after surgery. Specifically, patients were classified as “nicardipine (+)” if nicardipine infusion was continued during the VS period for up to 14 days after aSAH, unless discontinued earlier due to adverse events, such as hypotension. The infusion rate and dose titration were at the treating physician’s discretion; however, to be counted as “nicardipine (+),” the infusion had to be maintained at ≥1 mL/h while it was administered. Importantly, nicardipine and all other VS prophylactic medications were initiated postoperatively and used as prophylaxis, not reactively in response to symptoms (i.e., not as rescue therapy).

### 2.7. Variables and Outcomes for Modeling

Collected data included age, sex, history of hypertension (HT), diabetes mellitus, stroke, WFNS grade, aneurysm size and location, Fisher CT group [28], treatment modality (surgical clipping or endovascular coiling), periprocedural management, and VS prophylaxis during the VS period, including spinal/ventricular/cisternal drains, clazosentan, fasudil, cilostazol, statins, and nicardipine. Antiepileptic drug use (levetiracetam, lacosamide, perampanel) was also recorded. Radiological interventions for VS included intra-arterial fasudil and angioplasty. Cerebral complications (hemorrhage, edema, infarction, infection) and systemic complications (infections requiring antibiotics, syndrome of inappropriate secretion of antidiuretic hormone, lung edema, arrhythmia) were documented. Five outcomes were assessed, AVS, SVS, cerebral infarction, and mRS scores at discharge and at 6 months, dichotomized into good (0–2) or poor (3–6).

### 2.8. Statistical and Machine Learning Analyses 

Statistical comparisons, multivariable regression, machine learning analysis using Light Gradient Boosting Machine (LightGBM 4.6.0), and structural equation model (SEM) were performed to identify predictors and interactions. Detailed procedures, including SHapley Additive exPlanations (SHAP) interaction evaluation and SEM fit indices (χ^2^, root mean square error of approximation [RMSEA], comparative fit index [CFI], adjusted goodness-of-fit index [AGFI], and Tucker–Lewis Index [TLI]), are provided in Supplementary Method S2.

As a sensitivity analysis, we calculated absolute event rates across the four exposure strata (clazosentan ± × nicardipine ±) and additionally reported covariate-adjusted predicted probabilities estimated using the same covariates as those included in the corresponding multivariable models. We also repeated the multivariable analyses, adjusting for calendar time and including an indicator for the post-implementation period (after April 2022, when clazosentan became available in Japan) to account for potential time-related changes in practice (e.g., imaging frequency, intensive care unit pathways, and rehabilitation access). Calendar time was coded as a binary variable (pre- vs. post-April 2022).

## 3. Results

### 3.1. Patient Characteristics

Of the 1061 enrolled patients, 544 met the inclusion criteria and were included in the analysis (Figure 1). The mean age was 65.2 years, and 71.5% were female. The median WFNS grade was II, and the median Fisher group was 3. Endovascular coiling was performed in 48.2% of cases. Clazosentan was administered in 34.0%, fasudil in 81.8%, and nicardipine in 17.5% (Table 1). Overall, 20.6% developed AVS, 16.0% developed SVS, and 22.4% experienced cerebral infarction. Poor outcomes (mRS 3–6) were observed in 48.8% at discharge and in 33.7% (137/406) at 6 months (Table 2).

### 3.2. Risk Factors for AVS

**Figure 2:**
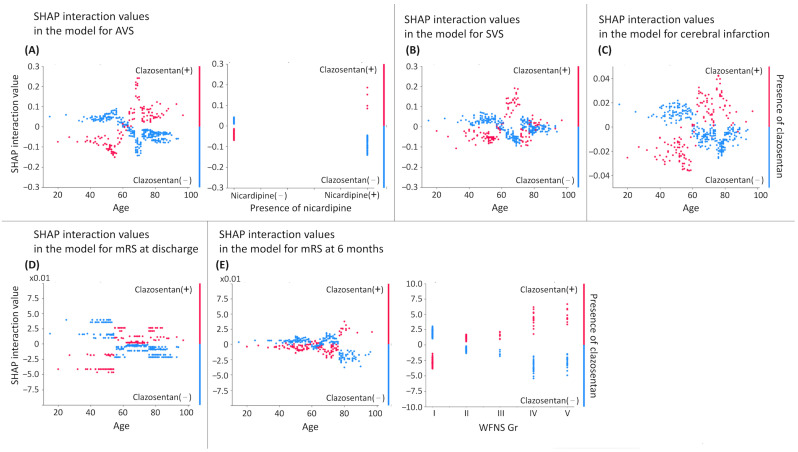
Interaction effects between clazosentan and other variables in LightGBM model. Interaction effects between clazosentan and other variables based on SHapley Additive exPlanations (SHAP) interaction values with clazosentan were derived from the Light Gradient Boosting Machine (LightGBM) model. Only those with negative correlations with the variables or whose sign varied depending on the variable values were visualized. Based on their distribution, interactions were created and incorporated into the multivariable analysis. Age and WFNS grades were treated as binary variables based on their SHAP interaction value distributions. (**A**) shows the interaction between clazosentan and age (**left**) and between nicardipine use and age (**right**) in the model for angiographic vasospasm (AVS). (**B**–**D**) show the interactions between clazosentan and age in models for symptomatic vasospasm (SVS), cerebral infarction, and the modified Rankin Scale (mRS) score at discharge, respectively. (**E**) displays the interactions between clazosentan and age (**left**) and WFNS grade (**right**) in the model for mRS score at 6 months. A higher SHAP interaction value (*y*-axis) indicates a greater likelihood of the outcome, implying a significant interaction effect. For example, regarding age in panel (**A**), the *x*-axis represents age. The color coding indicates clazosentan exposure, with red denoting the presence of clazosentan and blue denoting its absence. The *y*-axis shows the SHAP interaction value, which reflects the interaction-specific contribution of age × clazosentan to the model prediction. A positive SHAP interaction value indicates that the interaction increases the likelihood of the outcome (AVS), whereas values around zero indicate little interaction contribution, and negative values indicate a contribution toward a lower likelihood of the outcome. In this plot, among patients aged ≥60 years (*x*-axis ≥ 60) who received clazosentan (red), SHAP interaction values are predominantly positive, suggesting that the age × clazosentan combination increases the model-predicted probability of AVS. In contrast, among patients aged <60 years (*x*-axis < 60) who received clazosentan (red), SHAP interaction values tend to be negative, suggesting that the combination decreases the probability of AVS. Taken together, these patterns indicate an age-dependent interaction in which clazosentan is associated with a higher predicted probability of AVS in older patients (≥60 years) and a lower predicted probability in younger patients (<60 years). After this consideration of SHAP interaction in LightGBM, we subsequently re-examined it using multivariable regression to assess whether it remained statistically significant.

The LightGBM model achieved an area under the receiver operating characteristic curve (AUC) of 0.70. The variables with the three largest SHAP interaction values were age, HT, and nicardipine (see Supplementary Figure S1). Accordingly, these factors and their interactions with clazosentan were included in the multivariable analysis, in addition to significant variables from the univariable analysis. Age was treated as a binary variable based on the SHAP interaction value (left in Figure 2A). Multivariable analysis revealed that AVS was associated with the absence of HT (adjusted odds ratio [OR] for presence: 0.48; 95% confidence interval [CI] [0.28–0.83]), clazosentan use (OR for presence 0.27 [0.11–0.69]), and nicardipine (OR for presence 0.39 [0.19–0.80]), as well as the presence of cisternal drainage (OR 1.82 [1.06–3.13]). Co-administration of clazosentan and nicardipine showed a negative interaction, offsetting their individual protective effects (OR 1.85 [1.43–2.65]) (Table 3).

### 3.3. Risk Factors for SVS

The LightGBM model (AUC = 0.71) identified age, nicardipine use, and aneurysm size as top SHAP interaction variables (see Supplementary Figure S2). Age was dichotomized based on SHAP interaction (Figure 2B). In multivariable analysis, only the absence of clazosentan was associated with SVS (OR for presence 0.15, 95% CI [0.04–0.64]) (Table 4).

### 3.4. Risk Factors for Cerebral Infarction

The LightGBM model (AUC = 0.71) identified age, cisternal drainage, and statin use as the top SHAP interaction variables (see Supplementary Figure S5). Age was dichotomized based on SHAP interaction values (Figure 2C). Multivariable analysis showed that Fisher group 1–3 (OR 0.54, 95% CI [0.31–0.94]) and the presence of ventricular drainage (OR 1.74 [1.02–2.99]) were associated with cerebral infarction (see Supplementary Table S1).

### 3.5. Risk Factors for Poor Outcomes (mRS Score ≥ 3) at Discharge

Poor outcomes at discharge were observed in 48.8% of patients. The LightGBM model (AUC = 0.83) exhibited the highest SHAP interaction values for HT, Fisher group, and age (see Supplementary Figure S4). The Fisher CT group and age were binarized based on SHAP interaction (Figure 2D). AVS and SVS were strongly correlated (Spearman’s r = 0.862, *p* < 0.001; therefore, considering multicollinearity, only SVS was included in the multivariable analysis.

The multivariable analysis revealed that poor outcomes at discharge was associated with age 60 ≤ years (OR 3.56, 95% CI [2.02–6.26]), the presence of HT (OR 1.72 [1.04–2.87]), WFNS grade IV (OR 2.64 [1.42–4.93]) and V (OR 3.35 [1.62–6.96]), larger aneurysm size (OR 1.11 [1.03–1.19]), Fisher group 4 (OR 2.63 [1.42–4.91]), the presence of ventricular drainage (OR 2.84 [1.61–5.01]), and SVS (OR 3.57 [1.77–7.22]) (see Supplementary Table S2).

### 3.6. Risk Factors for Poor Outcomes (mRS Score ≥ 3) at 6 Months

Poor outcomes at 6 months were observed in 33.7% of patients. The LightGBM model (AUC = 0.84) identified aneurysm size, WFNS grade, and ventricular drainage as top SHAP interactions (see Supplementary Figure S5). Age and WFNS grades were treated as a binary variable considering their SHAP interaction value distribution (Figure 2E)

Multivariable analysis demonstrated that poor outcomes at 6 months were associated with age 60 ≤ years (OR 3.46, 95% CI [1.84–6.52]), the presence of HT (OR 1.79 [1.00–3.20]), larger aneurysm size (OR 1.15 [1.05–1.26]), Fisher group 4 (OR 2.27 [1.08–4.76]), the presence of ventricular drainage (OR 3.19 [1.56–6.53]), the absence of clazosentan (OR for presence 0.08 [0.01–0.68]), the presence of SVS (OR 2.43 [1.10–5.38]), cerebral complications (OR 2.99 [1.58–5.65]), and systemic complications (OR 2.33 [1.31–4.15]). In patients with WFNS grades II–V, clazosentan use was associated with an interaction effect towards poor prognosis (OR 7.49 [2.07–27.1]) (Table 5). Sensitivity analysis using WFNS grade as a categorical variable (see Supplementary Table S3) yielded similar directionality, supporting these findings.

### 3.7. Sensitivity Analyses for the Interaction Interpretation Between Clazosentan and Nicardipine

To facilitate interpretation of the interaction between clazosentan and nicardipine beyond the interaction OR in the multivariable analysis, we additionally examined outcomes across the four exposure strata defined by clazosentan completion (+/−) and nicardipine use (+/−). Absolute event rates for AVS, SVS, cerebral infarction, and poor functional outcomes (mRS ≥ 3) at discharge and 6 months are presented in Table 6.

In addition, we estimated covariate-adjusted predicted probabilities with 95% Wald CIs for each outcome, using the same covariates as in the corresponding multivariable models (Supplementary Table S4). For AVS, the combined-exposure stratum (clazosentan (+)/nicardipine (+)) did not differ significantly from either monotherapy stratum (all pairwise *p* > 0.05), whereas significant differences were observed among the other strata (*p* < 0.001). These marginal estimates indicate that the apparent interaction in the regression model should not be interpreted as evidence that the combined-exposure stratum is worse than monotherapy, particularly given the small size of the combined-exposure group.

We also repeated the multivariable analyses, including a calendar time indicator for the post-implementation period (post-April 2022, when clazosentan became available in Japan). The post-April 2022 period accounted for 56.1% of the cohort (*n* = 305). In these sensitivity analyses, the estimated associations were materially unchanged, and the calendar time indicator was not significantly associated with the outcomes in the corresponding models (Supplementary Tables S5–S10).

### 3.8. SEM of Relationships Between the Five Outcomes

**Figure 3:**
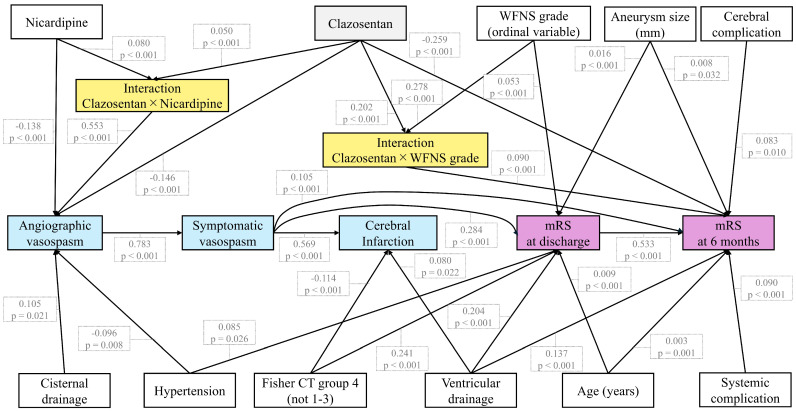
SEM in this study. Structural equation modeling (SEM) revealed that clazosentan reduced the incidence of angiographic vasospasm (AVS), which, in turn, decreased the occurrence of symptomatic vasospasm (SVS), resulting in better modified Rankin Scale (mRS) scores at discharge and 6 months. SEM also identified a direct effect of clazosentan on improving mRS scores at 6 months. Significant interactions between clazosentan and both the use of nicardipine and the World Federation of Neurosurgical Societies (WFNS) grade were observed. Only statistically significant paths are shown. Full results are provided in Supplementary Figure S6.

SEM (full paths in Supplementary Figure S6 and significant paths in Figure 3) exhibits good model fit (χ^2^: 161 [*p* = 0.172], RMSEA: 0.01, CFI: 0.997, AGFI: 0.958, and TLI: 0.996). Clazosentan reduced AVS incidence, which, in turn, decreased SVS occurrence, resulting in improved mRS scores at discharge and 6 months. Clazosentan’s direct effect on 6-month outcomes was identified. The interactions with nicardipine and WFNS grade were also confirmed. Overall, the SEM supports our findings in the multivariable analysis.

## 4. Discussion

In this retrospective study of 544 patients with aSAH across multiple Japanese centers, clazosentan use was associated with reduced AVS, SVS, and improved functional outcomes at 6 months. Furthermore, concomitant use of clazosentan with nicardipine appeared to increase the risk of AVS paradoxically.

### 4.1. Prophylactic Effect of Clazosentan on VS and Improved 6-Month Functional Outcomes

Clazosentan suppressed both AVS and SVS, consistent with findings from the CONSCIOUS trials [12,14] and Japanese RCT [19]. The higher mean age in our cohort (65.2 years) and the inclusion of patients often excluded from clinical trials—such as those with WFNS grade V [19], Fisher group 4 [19], or age ≥75 years [12,13,14,19] add real-world relevance to our results. Importantly, clazosentan was also associated with improved 6-month outcomes, consistent with a Japanese RCT [19] and a retrospective study [20].

The mechanism underlying this improvement remains unclear. While prevention of VS and secondary brain injury is likely a major contributor, clazosentan may also exert neuroprotective effects. Endothelin-1 functions as a pro-inflammatory cytokine, with its expression markedly increased under endothelial stress and in response to inflammatory cytokines and reactive oxygen species. Endothelin-1 is also abundant in inflammatory cells, reinforcing its role in neuroinflammation through ETA receptors [29]. As an ETA antagonist, clazosentan may reduce inflammation and provide neuroprotection, thereby contributing to favorable 6-month outcomes beyond its anti-VS effect.

Recent immune profiling work in chronic subdural hematoma has identified erythro-myeloid progenitors and unconventional natural killer T cells, highlighting that intracranial hemorrhage-related pathology can involve complex immune cell-driven cascades and microvascular dysregulation [30]. Although chronic subdural hematoma and aSAH are distinct entities, such findings support the broader concept that endothelin-1–mediated vasomotor effects and AVS represent only one component of a multifactorial inflammatory/immune milieu influencing delayed neurological injury.

### 4.2. Interaction Between Clazosentan and Nicardipine

In contrast to REACT trial [15], which reported no significant reduction in cerebral VS with clazosentan, our findings and those from Japanese studies [19,20,21] showed a protective effect. Key differences between REACT and our study include race (predominantly white vs. Asian, respectively), mean age (53.3 vs. 65.2 years), WFNS grades I–II (78.6% vs. 67.6%), rate of endovascular coiling (70.0% vs. 48.2%), and CCB use (95.5% vs. 17.5%; nicardipine in our study). Among these differences, we observed an attenuating interaction signal between clazosentan and nicardipine with respect to AVS. This finding should be interpreted cautiously because nicardipine infusion during the VS period may act as a marker of clinical severity or management complexity (e.g., hemodynamic instability, refractory HT), which could introduce confounding by indication despite adjustment for measured covariates. Nevertheless, our data raise the possibility that concomitant nicardipine use may modify the association between clazosentan and AVS prevention.

Evidence from prior trials provides context but does not establish causality for a drug–drug interaction. In the CONSCIOUS trials, intravenous nimodipine was prohibited, and only oral nimodipine was permitted [12,13,14]. Although the proportion of oral nimodipine use was not reported in CONSCIOUS-1, oral nimodipine was used in >90% of CONSCIOUS-2/3 patients. VS prevention was demonstrated only with the high dose of clazosentan (15 mg/h) in CONSCIOUS-3, whereas no significant VS prevention effect was observed with the lower doses of clazosentan in CONSCIOUS-2/3. Furthermore, in the Japanese RCT [19], where clazosentan reduced VS, intravenous nicardipine was prohibited. These differences may suggest that background vasoactive strategies may influence observed clazosentan-associated outcomes across studies; however, cross-trial comparisons remain indirect and susceptible to confounding.

Several mechanisms may explain this negative interaction, but these are only speculative. Clazosentan selectively inhibits ETA receptors, leaving Endothelin B (ETB) receptors active [31]. ETB signaling involves non-voltage-gated calcium channels (non-VGCCs), such as store-operated calcium channels (SOCCs), non-selective cation channels (NSCCs), and the Na^+^/Ca^2+^ exchanger (NCX). Nimodipine and nicardipine are voltage-dependent L-type CCB, which inhibits explicitly VGCC-mediated influx without affecting SOCC, NSCC, or NCX [32,33]. One hypothesis is that, under certain conditions, the combined use may trigger ETB-related influx, sustaining vasoconstriction and reducing the anti-spasm effect. Additionally, dual vasodilatory effects may cause hypotension, lower cerebral perfusion, and increase the risk of VS [34,35]. Accordingly, concomitant use may be less effective in some clinical contexts, particularly in susceptible patients, rather than being assumed to be harmful. Importantly, these proposed mechanisms are not directly tested in the present registry and should be regarded as hypothesis-generating.

Taken together, the observed interaction between clazosentan and nicardipine warrants further investigation in larger, blinded, prospective studies with standardized co-intervention protocols and richer hemodynamic and severity profiling to disentangle pharmacologic interaction from confounding by indication.

### 4.3. Interaction Between Clazosentan and WFNS Grade

In this study, the SHAP interaction value showed a strong interaction in severe WFNS grades, which aggravated the 6-month outcome, potentially due to the attenuated VS-preventive effect. A recent meta-analysis showed WFNS grades IV–V increased SVS risk [36]. Higher WFNS grades typically reflect larger hemorrhage volumes, elevated intracranial pressure, and reduced cerebral blood flow with a severe primary brain injury, which can be factors that cause cerebral VS. In particular, hemoglobin and its metabolite nitric oxide from hematoma have been implicated in VS development [37,38,39]. These factors may outweigh endothelin-1 in causing VS. Conversely, the therapeutic effect of clazosentan is likely to be more apparent in grade I cases where these complicated mechanisms are absent. This interaction may align with the Japanese RCT, which showed that grades I–II are more responsive than III–IV in reducing the risk of VS-related morbidity and all-cause mortality within 6 weeks [19].

Although re-analyses of CONSCIOUS-2/3 found clazosentan to be effective in thick and diffuse SAH [40], our findings contrarily suggest a reduced effect in severe WFNS grades, which may be accompanied by more SAH. Moreover, there are no interactions with the Fisher CT group regarding hematoma volume. The difference may stem from varying hematoma assessment methods. Further studies should clarify how WFNS and hematoma volume affect clazosentan efficacy.

### 4.4. Limitations

This study has some limitations. First, its retrospective design and modest sample size preclude definitive causal inference. Clazosentan administration was not randomized and depended on drug availability and physician discretion, raising the possibility of confounding by indication and selection bias. Such factors could influence both exposure assignment and subsequent outcomes, potentially biasing effect estimates despite multivariable adjustment. Treatment decisions were physician-dependent, and the unblinded design may have led to overestimation of clazosentan’s effect. The high use of fasudil in Japan (>80%) could also confound results, although adjustments were made. Moreover, VS prophylaxis was heterogeneous across patients, with frequent concomitant use of fasudil, cilostazol, statins, and drainage procedures; the timing and intensity of these co-interventions may have varied by clinical course and physician preference. Although we adjusted for major co-treatments where possible, residual confounding due to unmeasured or imperfectly captured treatment heterogeneity cannot be excluded. This residual confounding could produce an apparent “attenuation” effect. In addition, nicardipine infusion may have served as a severity marker rather than an independent competing exposure, and confounding by indication may have contributed to the observed interaction. Finally, missing data on VS and 6-month mRS further limit generalizability.

Second, while combining nonlinear machine learning models with classical regression and SEM provides a comprehensive analytic framework, interpretability and external validity should be approached with caution. Multiple testing also raises the possibility of Type I errors. Furthermore, inference regarding the clazosentan–nicardipine interaction remains limited because the combined-exposure stratum was small, as shown in the sensitivity analysis, leading to wide uncertainty in adjusted predicted probabilities and potentially unstable pairwise comparisons. Although adding a calendar time indicator (post-April 2022) did not materially change the regression estimates, a binary time variable may not fully capture secular changes, and residual time-related confounding cannot be completely excluded.

Third, the association between clazosentan and cerebral infarction was not confirmed, which may reflect the broad and non-specific nature of the infarction endpoint in our registry. Cerebral infarction was recorded without adjudication or subclassification into VS-attributable versus non-VS-related mechanisms (e.g., procedural/iatrogenic ischemia, thromboembolism, or hemodynamic causes), and detailed information required for attribution (timing relative to VS, symptom attribution, and serial imaging evolution) was not consistently available. Therefore, we could not perform a VS-attributable infarction subgroup analysis, and outcome misclassification may have diluted VS-specific associations and contributed to the discrepancy between cerebral infarction and SVS. Accordingly, cerebral infarction results should be interpreted as reflecting non-specific ischemic injury rather than a definitive VS-related infarction, and future studies should prospectively adjudicate VS-related infarction using stricter, mechanism-specific definitions.

Fourth, we assumed a linear progression from AVS to SVS to cerebral infarction. However, VS does not fully account for DCI, which is increasingly recognized as multifactorial, involving mechanisms such as cortical spreading depression and microthrombosis [41]. Moreover, clinical outcomes after cerebrovascular hemorrhage/ischemia are not determined by VS alone; they can be influenced by interacting anatomical and procedural factors (e.g., lesion complexity and tandem pathologies) [42], underscoring the limits of single-mechanism paradigms. This perspective further supports the use of integrative analytic frameworks such as interaction modeling and SEM in observational datasets. Finally, the observed interactions between clazosentan and nicardipine, and the WFNS grade, require validation in larger, blinded, prospective studies.

### 4.5. Clinical Takeaway

In this Japanese registry, clazosentan use was associated with a lower risk of AVS and SVS, supporting its role as a prophylactic option against VS after aSAH. The observed interaction signal with nicardipine should be regarded as hypothesis-generating and may reflect treatment context rather than a direct pharmacologic antagonism. In practice, clinicians should document concurrent vasoactive strategies and avoid assuming harm without prospective confirmation.

## 5. Conclusions

Clazosentan reduced AVS and SVS and improved 6-month mRS scores. The potential pharmacological interaction between clazosentan and nicardipine may adversely affect the prophylactic effectiveness of AVS, and clazosentan in WFNS grade I cases may improve 6-month outcomes.

## Figures and Tables

**Table 1:** Clinical characteristics and treatment of patients with SAH (*n* = 544).

	Overall (*n* = 544) (% or SD)	Clazosentan (+) (*n* = 185, 34.0%) (% or SD)	Clazosentan (−) (*n* = 359, 66.0%) (% or SD)
Age (years), mean (SD)	65.2 (14.2)	62.1 (13.7)	66.9 (14.3)
Sex (%female)	389 (71.5%)	130 (70.3%)	259 (72.1%)
Past history			
Hypertension (%)	224 (41.2%)	65 (35.1%)	159 (44.3%)
Diabetes mellitus (%)	60 (11.0%)	26 (14.1%)	34 (9.5%)
Stroke (%)	26 (4.8%)	3 (1.6%)	23 (6.4%)
WFNS grade			
I (%)	247 (45.4%)	83 (44.9%)	164 (45.7%)
II (%)	121 (22.2%)	50 (27.0%)	71 (19.8%)
III (%)	23 (4.2%)	11 (5.9%)	12 (3.3%)
IV (%)	79 (14.5%)	25 (13.5%)	54 (15.0%)
V (%)	74 (13.6%)	16 (8.6%)	58 (16.2%)
Aneurysm size (mm), mean (SD)	5.9 (3.5)	5.8 (2.9)	5.9 (2.5)
Aneurysm location			
ACA or ACoA (%)	168 (30.9%)	61 (33.0%)	107 (29.8%)
ICA (%)	188 (34.6%)	59 (31.9%)	129 (35.9%)
MCA (%)	127 (23.3%)	48 (25.9%)	79 (22.0%)
VA, BA, PCA, PICA (%)	61 (11.2%)	17 (9.2%)	44 (12.3%)
Fisher CT group			
1 (%)	10 (1.8%)	1 (0.5%)	9 (2.5%)
2 (%)	49 (9.0%)	16 (8.6%)	33 (9.2%)
3 (%)	362 (66.5%)	140 (75.7%)	222 (61.8%)
4 (%)	123 (22.6%)	28 (15.1%)	95 (26.5%)
Surgical procedure			
Endovascular coiling, not surgical clipping (%endovascular coiling)	262 (48.2%)	89 (48.1%)	173 (48.2%)
Spinal drainage (%)	196 (36.0%)	93 (50.3%)	103 (28.7%)
Ventricular drainage (%)	139 (25.6%)	49 (26.5%)	90 (25.1%)
Cisternal drainage (%)	95 (17.5%)	40 (21.6%)	55 (15.3%)
Cerebral vasospasm prophylaxis and other medications			
Fasudil (%)	445 (81.8%)	86 (46.5%)	359 (100%)
Cilostazol (%)	405 (74.4%)	142 (76.8%)	263 (73.3%)
Statin (%)	202 (37.1%)	105 (56.8%)	97 (27.0%)
Nicardipine (%)	95 (17.5%)	6 (3.2%)	89 (24.8%)
Antiepileptic drug (%)	219 (40.3%)	75 (40.5%)	144 (40.1%)

There were no missing values. Abbreviations; ACA, anterior cerebral artery; ACoA, anterior communicating artery; ICA, internal carotid artery; MCA, middle cerebral artery; SAH, subarachnoid hemorrhage; VA, vertebral artery; WFNS, World Federation of Neurosurgical Societies.

**Table 2:** Treatment outcomes of patients with SAH (*n* = 544).

	Overall (*n* = 544) (% or SD)	Clazosentan (+) (*n* = 185, 34.0%) (% or SD)	Clazosentan (−) (*n* = 359, 66.0%) (% or SD)
mRS score at discharge			
0 (%)	131 (24.1%)	50 (27.0%)	81 (22.6%)
1 (%)	86 (15.8%)	39 (21.1%)	47 (13.1%)
2 (%)	61 (11.2%)	26 (14.1%)	35 (9.7%)
3 (%)	61 (11.2%)	19 (10.3%)	42 (11.7%)
4 (%)	84 (15.4%)	18 (9.7%)	66 (18.4%)
5 (%)	93 (17.1%)	27 (14.6%)	66 (18.4%)
6 (%)	28 (5.1%)	6 (3.2%)	22 (6.1%)
mRS score at 6 months (*n* = 406)			
0 (%)	188/406 (46.3%)	97/159 (61.0%)	91/247 (36.8%)
1 (%)	52/406 (12.8%)	21/159 (13.2%)	31/247 (12.6%)
2 (%)	29/406 (7.1%)	7/159 (4.4%)	22/247 (8.9%)
3 (%)	30/406 (7.4%)	4/159 (2.5%)	26/247 (10.5%)
4 (%)	29/406 (7.1%)	10/159 (6.3%)	19/247 (7.7%)
5 (%)	41/406 (10.1%)	15/159 (9.4%)	26/247 (10.5%)
6 (%)	37/406 (9.1%)	5/159 (3.1%)	32/247 (13.0%)
Complications			
Angiographic vasospasm (%)	112 (20.6%)	28 (15.1%)	84 (23.4%)
Symptomatic vasospasm (%)	87 (16.0%)	19 (10.3%)	68 (18.9%)
IVR against cerebral vasospasm (%)	23 (4.2%)	5 (2.7%)	18 (5.0%)
Cerebral infarction (%)	122 (22.4%)	33 (17.8%)	89 (24.8%)
Cerebral complication (%)	215 (39.5%)	49 (26.5%)	166 (46.2%)
Systemic complication (%)	235 (43.2%)	49 (26.5%)	186 (51.8%)

Abbreviations: IVR, interventional radiology; mRS, modified Rankin Scale; SAH; subarachnoid hemorrhage.

**Table 3:** Multivariable logistic regression analysis of risk factors for angiographic vasospasm (*n* = 544).

	Univariable Analysis			Multivariable Analysis	
	Angiographic Vasospasm (−) (*n* = 432, 79.4%)	Angiographic Vasospasm (+) (*n* = 112, 20.6%)	*p* Values	OR (95% Confidence Interval)	*p* Values
Age; 60 ≤ years (%)	272 (63.0%)	70 (62.5%)	0.688	0.89 (0.51–1.52)	0.660
Interaction between clazosentan and age				1.47 (0.53–4.05)	0.457
Sex (%female)	307 (71.1%)	82 (73.2%)	0.653	1.13 (0.69–1.85)	0.641
Past history					
Hypertension (%)	189 (43.8%)	35 (31.3%)	0.017 *	0.48 (0.28–0.83)	0.008 **
Interaction between clazosentan and hypertension				1.44 (0.49–4.26)	0.511
Diabetes mellitus (%)	46 (10.6%)	14 (12.5%)	0.577		
Stroke (%)	22 (5.1%)	4 (3.6%)	0.498		
WFNS grade					
I (%)	202 (46.8%)	45 (40.2%)	0.185		
II (%)	98 (22.7%)	23 (20.5%)			
III (%)	20 (4.6%)	3 (2.7%)			
IV (%)	60 (13.9%)	19 (17.0%)			
V (%)	52 (12.0%)	22 (19.6%)			
Aneurysm size (mm), mean (SD)	5.9 (3.5)	6.1 (3.4)	0.349		
Aneurysm location			0.275		
ACA or ACoA (%)	133 (30.8%)	35 (31.3%)			
ICA (%)	148 (34.2%)	40 (35.7%)			
MCA (%)	97 (22.5%)	30 (26.8%)			
VA, BA, PCA, PICA (%)	54 (12.5%)	7 (6.2%)			
Fisher CT group					
1–3 (%)	342 (79.2%)	79 (70.5%)			
4 (%)	90 (20.8%)	33 (29.5%)	0.052		
Surgical procedure					
Endovascular coiling, not surgical clipping (%endovascular coiling)	210 (48.6%)	52 (46.4%)	0.680		
Spinal drainage (%)	147 (34.0%)	49 (43.8%)	0.056		
Ventricular drainage (%)	105 (24.3%)	34 (30.4%)	0.191		
Cisternal drainage (%)	67 (15.5%)	28 (25.0%)	0.018 *	1.82 (1.06–3.13)	0.030 *
Cerebral vasospasm prophylaxis and other medications					
Clazosentan (%)	157 (36.3%)	28 (25.0%)	0.024 *	0.27 (0.11–0.69)	0.006 **
Fasudil (%)	348 (80.6%)	97 (86.6%)	0.139	0.88 (0.37–2.09)	0.774
Cilostazol (%)	330 (76.4%)	75 (67.0%)	0.042 *	0.66 (0.41–1.05)	0.081
Statin (%)	160 (37.0%)	42 (37.5%)	0.928		
Nicardipine (%)	82 (19.0%)	13 (11.6%)	0.067	0.39 (0.19–0.80)	0.010 *
Interaction between clazosentan and nicardipine				1.85 (1.43–2.65)	0.002 **
Antiepileptic drug (%)	178 (41.2%)	41 (36.6%)	0.377		
Outcomes and complications					
mRS score 0–2 at discharge (%)	237 (54.9%)	41 (36.6%)	<0.001 ***		
mRS score 0–2 at 6 months (%) (*n* = 406)	228/322 (70.8%)	41/84 (48.8%)	0.002 **		
Symptomatic vasospasm (%)	0 (0%)	87 (77.7%)	<0.001 ***		
IVR against cerebral vasospasm (%)	3 (0.7%)	20 (17.9%)	<0.001 ***		
Cerebral infarction (%)	57 (13.2%)	65 (58.0%)	<0.001 ***		
Cerebral complication (%)	153 (35.4%)	62 (55.4%)	<0.001 ***		
Systemic complication (%)	177 (41.0%)	58 (51.8%)	0.040 *		

There were no missing values. In this multivariable analysis, we included the items that were significant in the univariable analysis, along with the three items with the largest SHapley Additive exPlanations interaction values in the machine learning model. Abbreviations: ACA, anterior cerebral artery; ACoA, anterior communicating artery; BA, basilar artery; ICA, internal carotid artery; IVR, interventional radiology; MCA, middle cerebral artery; mRS, modified Rankin Scale; OR, odds ratio; PCA, posterior cerebral artery; PICA, posterior inferior cerebellar artery; VA, vertebral artery; WFNS, World Federation of Neurosurgical Societies. *, *p* < 0.05; **, *p* < 0.01; ***, *p* < 0.001.

**Table 4:** Multivariable logistic regression analysis of risk factors for symptomatic vasospasm (*n* = 544).

	Univariable Analysis			Multivariable Analysis	
	Symptomatic Vasospasm (−) (*n* = 457, 84.0%)	Symptomatic Vasospasm (+) (*n* = 87, 16.0%)	*p* Values	OR (95% Confidence Interval)	*p* Values
Age; 60 ≤ years (%)	283 (61.9%)	59 (67.8%)	0.928	1.02 (0.57–1.83)	0.946
Interaction between clazosentan and age				1.35 (0.42–4.41)	0.616
Sex (%female)	325 (71.1%)	64 (73.6%)	0.782	1.03 (0.60–1.75)	0.923
Past history					
Hypertension (%)	196 (42.9%)	28 (32.2%)	0.051		
Diabetes mellitus (%)	49 (10.7%)	11 (12.5%)	0.631		
Stroke (%)	22 (4.8%)	4 (4.5%)	0.907		
WFNS grade					
I (%)	212 (46.4%)	35 (39.8%)	0.089		
II (%)	106 (23.2%)	15 (17.0%)			
III (%)	20 (4.4%)	3 (3.4%)			
IV (%)	63 (13.8%)	16 (18.2%)			
V (%)	55 (12.1%)	19 (21.6%)			
Aneurysm size (mm), mean (SD)	5.8 (3.5)	6.4 (3.6)	0.105	1.02 (0.95–1.09)	0.559
Interaction between clazosentan and aneurysm size				1.07 (0.90–1.28)	0.455
Aneurysm location			0.318		
ACA or ACoA (%)	144 (31.5%)	24 (27.6%)			
ICA (%)	156 (34.2%)	32 (36.8%)			
MCA (%)	102 (22.3%)	25 (28.7%)			
VA, BA, PCA, PICA (%)	55 (12.0%)	6 (6.9%)			
Fisher CT group					
1–3 (%)	358 (78.3%)	63 (72.4%)			
4 (%)	99 (21.7%)	24 (27.6%)	0.253		
Surgical procedure					
Endovascular coiling, not surgical clipping (%endovascular coiling)	222 (48.6%)	40 (46.0%)	0.579		
Spinal drainage (%)	159 (34.9%)	37 (42.5%)	0.199		
Ventricular drainage (%)	107 (23.5%)	32 (36.8%)	0.011 *	1.31 (0.72–2.38)	0.375
Cisternal drainage (%)	71 (15.6%)	24 (27.6%)	0.008 **	1.80 (0.92–3.51)	0.082
Cerebral vasospasm prophylaxis and other medications					
Clazosentan (%)	166 (36.4%)	19 (21.6%)	0.007 **	0.15 (0.04–0.64)	0.010 *
Fasudil (%)	369 (80.7%)	76 (87.4%)	0.226	0.52 (0.19–1.46)	0.215
Cilostazol (%)	346 (75.7%)	59 (67.8%)	0.082	0.71 (0.42–1.18)	0.188
Statin (%)	168 (36.8%)	34 (39.1%)	0.750		
Nicardipine (%)	85 (18.6%)	10 (11.4%)	0.001 **	0.47 (0.21–1.01)	0.052
Interaction between clazosentan and nicardipine				3.84 (0.36–41.10)	0.265
Antiepileptic drug (%)	189 (41.4%)	30 (34.5%)	0.198		
Outcomes and complications (%)					
mRS score 0–2 at discharge (%)	260 (56.9%)	18 (20.5%)	<0.001 ***		
mRS score 0–2 at 6 months (%) (*n* = 406)	245/339 (72.3%)	43/67 (64.2%)	<0.001 ***		
Angiographic vasospasm (%)	24 (5.3%)	87 (100%)	<0.001 ***		
IVR against cerebral vasospasm (%)	3 (0.7%)	20 (23.0%)	<0.001 ***		
Cerebral infarction (%)	60 (13.2%)	62 (71.3%)	<0.001 ***		
Cerebral complication (%)	164 (36.0%)	51 (58.6%)	<0.001 ***		
Systemic complication (%)	190 (41.6%)	45 (51.7%)	0.101		

There were no missing values. In this multivariable analysis, we included the items that were significant in the univariable analysis, along with the three items with the largest SHapley Additive exPlanations interaction values in the machine learning model. Abbreviations: ACA, anterior cerebral artery; ACoA, anterior communicating artery; BA, basilar artery; ICA, internal carotid artery; IVR, interventional radiology; MCA, middle cerebral artery; mRS, modified Rankin Scale; OR, odds ratio; PCA, posterior cerebral artery; PICA, posterior inferior cerebellar artery; VA, vertebral artery; WFNS, World Federation of Neurosurgical Societies; *, *p* < 0.05; **, *p* < 0.01; ***, *p* < 0.001.

**Table 5:** Multivariable logistic regression analysis of risk factors for poor outcomes (mRS score ≥ 3) at 6 months (*n* = 406).

	Univariable Analysis			Multivariable Analysis	
	mRS Score 0–2 at 6 Months (*n* = 269, 66.3%)	mRS Score 3–6 at 6 Months (*n* = 137, 33.7%)	*p* Values	OR (95% Confidence Interval)	*p* Values
Age; 60 ≤ years (%)	170 (63.2%)	118 (86.1%)	<0.001 ***	3.46 (1.84–6.52)	<0.001 ***
Sex (%female)	194 (72.1%)	99 (72.3%)	0.976	0.80 (0.43–1.48)	0.472
Past history					
Hypertension (%)	95 (35.3%)	63 (46.0%)	0.037 *	1.79 (1.00–3.20)	0.049 *
Diabetes mellitus (%)	35 (13.0%)	16 (11.7%)	0.702		
Stroke (%)	5 (1.9%)	8 (5.8%)	0.032 *	2.00 (0.39–10.16)	0.403
WFNS grade II–V compared to I as a reference			0.349	0.77 (0.41–1.46)	0.433
Interaction between clazosentan and WFNS grade II–V				7.49 (2.07–27.10)	0.002 **
Aneurysm size (mm), mean (SD)	5.5 (2.5)	6.9 (4.7)	<0.001 ***	1.15 (1.05–1.26)	0.003 **
Interaction between clazosentan and aneurysm size				0.99 (0.79–1.24)	0.968
Aneurysm location			0.479		
ACA or ACoA (%)	78 (29.0%)	48 (35.0%)			
ICA (%)	100 (37.2%)	41 (29.9%)			
MCA (%)	63 (23.4%)	33 (24.1%)			
VA, BA, PCA, PICA (%)	28 (10.4%)	15 (11.0%)			
Fisher CT group					
1–3 (%)	237 (88.1%)	99 (72.3%)			
4 (%)	32 (11.9%)	38 (27.7%)	<0.001 ***	2.27 (1.08–4.76)	0.030 *
Surgical procedure					
Endovascular coiling, not surgical clipping (%endovascular coiling)	131 (48.7%)	58 (42.3%)	0.224		
Spinal drainage (%)	125 (46.5%)	51 (37.2%)	0.076		
Ventricular drainage (%)	54 (20.1%)	58 (42.3%)	<0.001 ***	3.19 (1.56–6.53)	0.001 **
Interaction between clazosentan and ventricular drainage				2.79 (0.72–10.70)	0.137
Cisternal drainage (%)	49 (18.2%)	36 (26.3%)	0.059		
Cerebral vasospasm prophylaxis and other medications					
Clazosentan (%)	125 (46.5%)	34 (24.8%)	<0.001 ***	0.08 (0.01–0.68)	0.020 *
Fasudil (%)	201 (74.7%)	116 (84.7%)	0.022 *	0.47 (0.15–1.41)	0.177
Cilostazol (%)	194 (72.1%)	96 (70.1%)	0.666		
Statin (%)	116 (43.1%)	66 (48.2%)	0.330		
Nicardipine (%)	55 (20.4%)	25 (18.2%)	0.599		
Antiepileptic drug (%)	127 (47.2%)	54 (39.4%)	0.135		
Outcomes and complications					
Angiographic vasospasm (%)	41 (15.2%)	43 (31.4%)	<0.001 ***	†	
Symptomatic vasospasm (%)	24 (8.9%)	43 (31.4%)	<0.001 ***	2.43 (1.10–5.38)	0.029 *
IVR against cerebral vasospasm (%)	12 (4.5%)	9 (6.6%)	0.355		
Cerebral infarction (%)	48 (17.8%)	54 (39.4%)	<0.001 ***	1.73 (0.83–3.59)	0.141
Cerebral complication (%)	49 (18.2%)	60 (43.8%)	<0.001 ***	2.99 (1.58–5.65)	<0.001 ***
Systemic complication (%)	71 (26.4%)	71 (51.8%)	<0.001 ***	2.33 (1.31–4.15)	0.004 **

There were no missing values. In this multivariable analysis, we included the items that were significant in the univariable analysis, along with the three items with the largest SHapley Additive exPlanations interaction values in the machine learning model. Abbreviations: ACA, anterior cerebral artery; ACoA, anterior communicating artery; BA, basilar artery; ICA, internal carotid artery; IVR, interventional radiology; MCA, middle cerebral artery; mRS, modified Rankin Scale; OR, odds ratio; PCA, posterior cerebral artery; PICA, posterior inferior cerebellar artery; VA, vertebral artery; WFNS, World Federation of Neurosurgical Societies; *, *p* < 0.05; **, *p* < 0.01; ***, *p* < 0.001. †; Presence of angiographic and symptomatic vasospasm has a strong correlation (r = 0.862, *p* < 0.001 by Spearman’s correlation coefficient), so only symptomatic vasospasm was included in the multivariable analysis, considering the multicollinearity.

**Table 6:** Absolute event rates for clinical outcomes by exposure strata.

Exposure Stratum	AVS	SVS	Cerebral Infarction	mRS ≥ 3 at Discharge	mRS ≥ 3 at 6 Months
Clazosentan (+)/Nicardipine (+)	3/6 (50.0%)	1/6 (16.7%)	2/6 (33.3%)	2/6 (33.3%)	2/6 (33.3%)
Clazosentan (+)/Nicardipine (−)	25/179 (14.0%)	18/179 (10.1%)	31/179 (17.3%)	68/179 (38.0%)	32/153 (20.9%)
Clazosentan (−)/Nicardipine (+)	10/89 (11.2%)	9/89 (10.1%)	22/89 (24.7%)	46/89 (51.7%)	23/74 (31.1%)
Clazosentan (−)/Nicardipine (−)	74/270 (27.4%)	59/270 (21.9%)	67/270 (24.8%)	150/270 (55.6%)	80/173 (46.2%)

Abbreviations: AVS, angiographic vasospasm; mRS, modified Rankin Scale; SVS, symptomatic vasospasm.

## Data Availability

De-identified data are available from the corresponding author upon reasonable request in accordance with the policy of the Shimane Prefectural Central Hospital Review Board.

## Supplementary Materials

The following supporting information can be downloaded at: https://www.mdpi.com/article/10.3390/jcm15041383/s1, Supplementary Method S1. Details about the study protocol of DCI-Japan; Supplementary Method S2. Detailed methods [43,44,45,46,47]; Figure S1. Summary plot of SHapley Additive exPlanations (SHAP) feature importance in the model predicting the occurrence of angiographic vasospasm (A). Red dots indicate high feature values (e.g., older age) for that individual patient, whereas blue dots indicate low underlying feature values (e.g., younger age). For binary categorical features, blue dots indicate its absence, and red dots indicate its presence. Visualizing patient-specific SHAP values for each feature directly shows how each feature’s values (dot color) relate to its impact on predicted model output (left or right shift on the x-axis). The SHAP interaction values are summarized (B). Figure S2. Summary plot of SHapley Additive exPlanations (SHAP) feature importance in the model predicting the occurrence of symptomatic vasospasm (A). Red dots indicate high feature values (e.g., older age) for that individual patient, whereas blue dots indicate low underlying feature values (e.g., younger age). For binary categorical features, blue dots indicate its absence, and red dots indicate its presence. Visualizing patient-specific SHAP values for each feature directly shows how each feature’s values (dot color) relate to its impact on predicted model output (left or right shift on the x-axis). The SHAP interaction values are summarized (B). Figure S3. Summary plot of SHapley Additive exPlanations (SHAP) feature importance in the model predicting the occurrence of cerebral infarction (A). Red dots indicate high feature values (e.g., older age) for that individual patient, whereas blue dots indicate low underlying feature values (e.g., younger age). For binary categorical features, blue dots indicate its absence, and red dots indicate its presence. Visualizing patient-specific SHAP values for each feature directly shows how each feature’s values (dot color) relate to its impact on predicted model output (left or right shift on the x-axis). The SHAP interaction values are summarized (B). Figure S4. Summary plot of SHapley Additive exPlanations (SHAP) feature importance in the model predicting the modified Rankin Scale (mRS) score 3–6 at discharge (A). Red dots indicate high feature values (e.g., older age) for that individual patient, whereas blue dots indicate low underlying feature values (e.g., younger age). For binary categorical features, blue dots indicate its absence, and red dots indicate its presence. Visualizing patient-specific SHAP values for each feature directly shows how each feature’s values (dot color) relate to its impact on predicted model output (left or right shift on the x-axis). The SHAP interaction values are summarized (B). Figure S5. Summary plot of SHapley Additive exPlanations (SHAP) feature importance in the model predicting the modified Rankin Scale (mRS) score 3–6 at 6 months (A). Red dots indicate high feature values (e.g., older age) for that individual patient, whereas blue dots indicate low underlying feature values (e.g., younger age). For binary categorical features, blue dots indicate its absence, and red dots indicate its presence. Visualizing patient-specific SHAP values for each feature directly shows how each feature’s values (dot color) relate to its impact on predicted model output (left or right shift on the x-axis). The SHAP interaction values are summarized (B). Figure S6. The full paths of structural equation modeling (SEM, Figure 3) revealed that clazosentan reduced the incidence of angiographic vasospasm, which, in turn, decreased the occurrence of symptomatic vasospasm, ultimately leading to improved modified Rankin Scale (mRS) scores at discharge and 6 months. It also showed a direct effect of clazosentan on improving the mRS score at 6 months. Significant interactions between clazosentan and both nicardipine use and World Federation of Neurosurgical Societies (WFNS) grade were also confirmed. Table S1. Multivariable logistic regression analysis of risk factors for cerebral infarction (*n* = 544). Table S2. Multivariable logistic regression analysis of risk factors for poor outcomes (mRS score ≥ 3) at discharge (*n* = 544). Table S3. Multivariable logistic regression analysis of risk factors for poor outcomes (mRS score ≥ 3) at 6 months (*n* = 406). Table S4. Multivariable logistic regression–derived adjusted predicted probabilities for AVS, SVS, cerebral infarction, and mRS outcomes according to clazosentan and nicardipine use. Table S5. Multivariable logistic regression analysis of risk factors for angiographic vasospasm (sensitivity analysis). (*n* = 544) Table S6. Multivariable logistic regression analysis of risk factors for symptomatic vasospasm (sensitivity analysis) (*n* = 544). Table S7. Multivariable logistic regression analysis of risk factors for cerebral infarction (sensitivity analysis) (*n* = 544). Table S8. Multivariable logistic regression analysis of risk factors for poor outcomes (mRS score ≥ 3) at discharge (sensitivity analysis) (*n* = 544). Table S9. Multivariable logistic regression analysis of risk factors for poor outcomes (mRS score ≥ 3) at 6 months (sensitivity analysis) (*n* = 406). Table S10. Multivariable logistic regression analysis of risk factors for poor outcomes (mRS score ≥ 3) at 6 months (sensitivity analysis) (*n* = 406).

## Abbreviations

The following abbreviations are used in this manuscript:

ACA	anterior cerebral artery
ACoA	anterior communicating artery
AGFI	adjusted goodness-of-fit index
aSAH	aneurysmal subarachnoid hemorrhage
AUC	area under the curve
AVS	angiographic vasospasm
BA	basilar artery
CCB	calcium channel blocker
CFI	comparative fit index
CI	confidence interval
CT	computed tomography
DCI	delayed cerebral ischemia
DCI Japan	Database of Cohort Study for Outcome of SAH In Japan
ETA	Endothelin A
ETB	Endothelin B
Fasudil	fasudil hydrochloride hydrate
HT	hypertension
ICA	internal carotid artery
IVR	Interventional radiology
LightGBM	Light Gradient Boosting Machine
MCA	middle cerebral artery
mRS	modified Rankin Scale
NCX	Na^+^/Ca^2+^ exchanger
NSCC	non-selective cation channel
OR	odds ratio
PCA	posterior cerebral artery
PICA	posterior inferior cerebellar artery
RCT	randomized controlled trial
RMSEA	root mean square error of approximation
Q1–Q3	25th–75th percentile
SAH	subarachnoid hemorrhage
SD	standard deviation
SEM	structural equation modeling
SHAP	SHapley Additive exPlanations
SOCC	store-operated calcium channel
SVS	symptomatic vasospasm
TLI	Tucker–Lewis Index
VA	vertebral artery
VGCC	voltage-gated calcium channel
VS	vasospasm
WFNS	World Federation of Neurosurgical Surgeons
