# Supplementary material for: Machine Learning-Identified Potential Interaction Between Clazosentan and Nicardipine in Patients with Subarachnoid Hemorrhage
Source: J Clin Med. 2026 Feb 10;15(4):1383. doi: 10.3390/jcm15041383 (PMC12941893; doi:10.3390/jcm15041383)
Supplement: Supplementary file 1 [file jcm-15-01383-s001.zip › JCM_SupMethod S1__251227.pdf]

Supplementary Method S1. Details about the study protocol of DCI-Japan

PROTOCOL TITLE: DATABASE OF COHORT STUDY FOR OUTCOME OF SAH IN JAPAN (DCI JAPAN)

TYPE OF RESEARCH: Observational

PROTOCOL VERSION: VERSION.1.3

PROTOCOL VERSION DATE: 2024.4.1

Table of Contents

1. INTRODUCTION

1.1 Background and Rationale

1.2 Objectives

2. STUDY DESIGN

2.1 Type of Study

2.2 Study Population

2.3 Duration of Study

3. TARGET STUDY POPULATION

3.1 Number of Participants

3.2 Gender, Age, Racial, and Ethnic Origin of Participants

3.3 Inclusion Criteria

3.4 Exclusion Criteria

3.5 Vulnerable Participants

3.6 Identification and Recruitment

3.7 Location

3.8 Participant Payments

4. DATA HANDLING AND RECORD KEEPING

4.1 Data Capture, Management, & Collection Procedures

4.2 Secure Storage Data

4.3 Confidentiality

4.4 Unanticipated Problem Reporting

5. RISKS AND BENEFITS

5.1 Risks

5.2 Likelihood of Occurrence

5.3 Benefits to Participants/Others

6. INFORMED CONSENT

7. STATISTICAL PLAN

7.1 Sample Size Determination

7.2 Statistical Methods

8. ETHICAL CONSIDERATIONS

8.1 Conflict of Interest

9. REFERENCES

LIST OF ABBREVIATIONS

angiographic vasospasm (AVS); cerebral infarction (CI); computed tomography (CT); delayed cerebral ischemia (DCI) ; “Database of Cohort Study for Outcome of SAH In Japan” (DCI Japan); digital subtraction angiography(DSA); magnetic resonance imaging (MRI); modified Rankin Scale (mRS); Subarachnoid hemorrhage (SAH); structural equation modeling (SEM); symptomatic vasospasm (SVS)

1. INTRODUCTION

1.1 BACKGROUND AND RATIONALE

Subarachnoid hemorrhage (SAH) remains a serious form of stroke with limited improvement in outcomes.[1][4] Among the major challenges in SAH management is delayed cerebral ischemia (DCI), a condition now recognized as multifactorial. While large-vessel narrowing and reduced blood flow are key mechanisms, recent insights point to early brain injury, microcirculatory dysfunction, loss of autoregulation, cortical spreading depolarization, and microthrombosis as additional contributors.[2]Effective strategies for preventing and treating DCI remain lacking. The DCI Japan database was established to comprehensively evaluate treatment practices and identify clinical factors that influence outcomes in patients with aneurysmal SAH.

1.2 OBJECTIVES

Objectives

To clarify the factors affecting DCI, its prevention and treatment, and overall outcome after aneurysmal SAH.

2. STUDY DESIGN

2.1 Type of Study

Prospective and retrospective observational cohort study without use of human samples.

2.2 Study Population

All hospitalized patients diagnosed with SAH during the prescribed period.

2.3 Duration of Study

- Patient enrollment: January 1, 2000 to December 31, 2030
- Study period: From IRB approval until December 31, 2032

3. Target Study Population

3.1 Number of Participants

Approximately 40,000 patients with aneurysmal SAH.

3.2 Sex, Age, Racial, and Ethnic Origin of Participants

No restrictions.

3.3 Inclusion Criteria

- Patients with SAH on admission diagnosed through computed tomography (CT), magnetic resonance imaging (MRI), or cerebrospinal fluid findings
- Presence of cerebral aneurysm confirmed by CT angiography, magnetic resonance angiography, or digital subtraction angiography (DSA)

3.4 Exclusion Criteria

- No evidence of SAH

- No cerebral aneurysm confirmed by CT angiography, magnetic resonance angiography, or DSA

3.5 Vulnerable Participants

Children under 18 years, prisoners, and other vulnerable populations were excluded.

3. 6 Participant Identification and Recruitment

Consecutive patients with aneurysmal SAH are identified from hospital admission records at each participating institution. Each institution assigns a responsible investigator—typically a board-certified neurosurgeon—to oversee case identification and data entry. All registered cases are consecutively enrolled in principle, following standardized protocols.

3. 7 Location

Ten high-volume cerebrovascular centers in Japan. A high-volume center is defined as a hospital with more than 300 beds, an independent ICU, and the capability to perform surgery for subarachnoid hemorrhage. Additionally, the hospital must conduct more than 300 neurosurgical procedures annually and have neurosurgeons, interventional neuroradiologists, and neuroradiologists consistently available. The 10 participating facilities are as follows:

- Shimane Prefectural Central Hospital
- Hiroshima University
- Japanese Red Cross Asahikawa Hospital

- Kyorin University
- Kurashiki Central Hospital
- Kurume University School of Medicine
- Saitama Medical University International Medical Center
- Tokai University
- Japan Red Cross Medical Center
- Fujita Health University School of Medicine

3. 8 Participant Payments

Not applicable.

4. DATA HANDLING AND RECORD-KEEPING

4.1 Data Capture, Management, & Collection Procedures

The database is entered by the person responsible for each institution after blind double-checked by board-certified neurosurgeons in Japan. De-identified data is then shared via secure, password-protected cloud storage. Central data integration and analysis are performed by Shimane Prefectural Central Hospital.

4.2 Secure Storage of Data

Files are stored in secure hospital servers and locked cabinets. Retention for 3 years after publication, then anonymized disposal.

4.3 Confidentiality

Anonymization is ensured by assigning codes. Only the principal investigator manages the correspondence table.

4.4 Unanticipated Problems Reporting

The principal investigator will report issues affecting ethical or scientific validity to the hospital director without delay.

.

5. Risks and Benefits

5.1 Risks

None. No intervention is involved.

5.2 Likelihood of Occurrence

None.

5.3 Benefits to Participants/Others

Future improvements in SAH care and research.

6. Informed Consent

Consent is waived due to retrospective design but opt-out procedures and public notices are implemented per ethical guidelines.

7. Statistical Plan and Considerations

7.1 Sample Size Determination

Approximately 40,000 SAH cases, no statistical power calculation was conducted.

7.2 Statistical Methods

This study collects data on demographics, pre-morbid functional status, aneurysm characteristics, treatment modalities, medications, complications, and outcomes (e.g., mRS at discharge and at 6 months). Multivariable logistic regression will be used to identify independent risk factors for key outcomes, including delayed cerebral ischemia, angiographic vasospasm (AVS), symptomatic vasospasm (SVS), and cerebral infarction (CI). The classification into three VS-related events followed the classic model of vasoconstriction leading to ischemic symptoms.[3]

Definitions:

- AVS: Defined as ≥50% arterial narrowing on DSA or CTA in any cerebral vascular territory, compared with preoperative imaging. Narrowing must not be due to atherosclerosis, catheter-induced spasm, or vessel hypoplasia, and is diagnosed solely based on radiologic findings, regardless of symptoms.[5]
- SVS: Defined as new focal neurological deficits or a decrease of ≥2 points in the Glasgow Coma Scale, deemed to be caused by ischemia due to vasospasm after excluding other potential causes such as hydrocephalus, seizures, metabolic derangement, infection, or oversedation. SVS in this study satisfies both the AVS and DCI definitions.[5]

- CI: Defined as new infarcts detected by CT or MRI within 6 weeks of SAH onset, or confirmed at autopsy, not present on imaging performed 24–48 h after early aneurysm occlusion, and not attributable to surgical procedures or other causes. Hypodensities from ventricular catheters or intraparenchymal hematomas are excluded. CI includes infarcts not necessarily caused by vasospasm, such as cardioembolic or atherothrombotic infarctions.[5]

Statistical significance is defined as $p < 0.05$ (two-sided).

8. Ethical Considerations

Study approved by the Shimane Prefectural Central Hospital Ethics Committee (Approval No. R22-020), following the Declaration of Helsinki and STROBE guidelines.

8.1 Conflict of Interest

None declared.

9. References

1. Daou BJ, Koduri S, Thompson BG, Chaudhary N, Pandey AS (2019) Clinical and experimental aspects of aneurysmal subarachnoid hemorrhage. CNS Neurosci Ther 25:1096–1112. doi: 10.1111/cns.13222

2. Francoeur CL, Mayer SA (2016) Management of delayed cerebral ischemia after subarachnoid hemorrhage. Crit Care 20:277. doi: 10.1186/s13054-016-1447-6

3. Macdonald RL (2013) Does Prevention of Vasospasm in Subarachnoid Hemorrhage Improve Clinical Outcome? Yes. Stroke 44:S31–S33. doi: 10.1161/STROKEAHA.112.679142

4. Roquer J, Cuadrado-Godia E, Guimaraens L, Conesa G, Rodríguez-Campello A, Capellades J, García-Arnillas MP, Fernández-Candil JL, Avellaneda-Gómez C, Giralt-Steinhauer E, Jiménez-Conde J, Soriano-Tárraga C, Villalba-Martínez G, Vivanco-Hidalgo RM, Vivas E, Ois A (2020) Short- and long-term outcome of patients with aneurysmal subarachnoid hemorrhage. Neurology 95:e1819–e1829. doi: 10.1212/WNL.0000000000010618

5. Vergouwen MDI, Vermeulen M, van Gijn J, Rinkel GJE, Wijdicks EF, Muizelaar JP, Mendelow AD, Juvela S, Yonas H, Terbrugge KG, Macdonald RL, Diringer MN, Broderick JP, Dreier JP Definition of Delayed Cerebral Ischemia After Aneurysmal Subarachnoid Hemorrhage as an Outcome Event in Clinical Trials and Observational Studies
