# Supplementary material for: Machine Learning-Identified Potential Interaction Between Clazosentan and Nicardipine in Patients with Subarachnoid Hemorrhage
Source: J Clin Med. 2026 Feb 10;15(4):1383. doi: 10.3390/jcm15041383 (PMC12941893; doi:10.3390/jcm15041383)
Supplement: Supplementary file 1 [file jcm-15-01383-s001.zip › JCM_SupMethod S2_251227.pdf]

Supplementary Method S2. Detailed methods

Statistical and machine learning analysis

Continuous variables with normal distribution were described as means (standard deviation [SD]) and compared using a t-test. Continuous variables without normal distribution and ordinal variables were described as medians (25th–75th percentile; Q1–Q3) and compared using the Mann-Whitney U test. Categorical variables were described as actual numbers and percentages, and the proportions were compared using chi-square (χ^2) or Fisher's exact tests. There was no missing data. Significant variables in univariable analyses were used for multivariable analysis. No statistical power calculation was conducted before the study, and the sample size was based on available data.

To identify the factors that interact with clazosentan, five prediction models were developed for each outcome using a supervised machine learning approach with a Light Gradient Boosting Machine (LightGBM) and the abovementioned variables. To ensure robust performance, the dataset was split, with 80% allocated to the training dataset and 20% reserved for independent model evaluation. To address class imbalances in the outcome variable, the Synthetic Minority Oversampling Technique was employed to augment the dataset before training. A ten-fold cross-validation approach was implemented during training to further enhance model reliability and avoid overfitting. Hyperparameter tuning was performed using a random grid search. After developing the model based solely on the training dataset, we tested its performance using a test dataset that was not used for model training or tuning. The predictive capabilities of the model were quantified using the area under the receiver operating characteristic curve (AUC). To improve interpretability, SHapley Additive exPlanations (SHAP) values were computed to elucidate the feature importance for each prediction. Additionally, SHAP interaction values were calculated to analyze and quantify the interaction effects between features. The three items with the largest SHAP interaction values were entered into the multivariable analysis as interactions. The SHAP interaction values with clazosentan derived from the Light Gradient Boosting Machine (LightGBM) models were examined (**Figure 2**). For those that did not show a positive correlation with the variables or whose sign varied depending on the variable values, the SHAP interaction values were visualized. Based on their visualized distribution, interactions were created and incorporated into the multivariable analysis.

A multivariable logistic regression model with treatment institution as a random effect (generalized linear mixed model) was used to investigate the association between each outcome and the factors extracted through the procedures described above. In this multivariable analysis, we included the items that were significant in the univariable analysis, along with the three items with the largest SHAP interaction values in the machine learning model. Interactions were entered as the

product of the presence of clazosentan and the item. The number of variables used in the multivariable analysis was limited to approximately 1/10th of the sample size for each outcome. If there were variables with a correlation coefficient of 0.8 or higher and suggested high multicollinearity, one was selected for use based on clinical reasonability. Odds ratios (ORs) and 95% confidence intervals (CIs) of items were determined.

SEM was performed to examine the relationships between the significant variables revealed by the multivariable analysis and the five outcomes. List-wise deletions were used for cases without mRS score at 6 months. The model was estimated using the restricted maximum likelihood method. The performance indexes included χ^2, root mean square error of approximation (RMSEA), comparative fit index (CFI), adjusted goodness-of-fit index (AGFI), and Tucker-Lewis Index (TLI).

Statistical significance was set at a two-tailed $p < 0.05$. SPSS Statistics version 29.0.0 (IBM Corp., Armonk, NY, USA), Python 3.9.0, Pandas 2.0–2, PyCaret 3.1.0, SHAP 0.46.0, SHAP-IQ 1.1.1, semopy 2.0, and Matplotlib 3.5.1 were used.

This approach—narrowing down variables and interactions using machine learning, followed by validation through multivariable analysis—has been reported in previous studies [1–5].

1. Fukuda H, Hyohdoh Y, Kawada K, Sorimachi T, Suzuki K, Kurita H, Uezato M, Chin M, Okada K, Nakatomi H, Shiokawa Y, Ishikawa T, Kawamata T, Morioka J, Nakahara I, Shimamura N, Ohkuma H, Ichihara N, Ueba T, Ikawa F (2025) Risk factors of short-term poor functional outcomes and long-term durability of ruptured large or giant intracranial aneurysms. J Neurosurg 1–10. doi: 10.3171/2024.8.JNS24894

2. Johnsen PV, Riemer-Sørensen S, DeWan AT, Cahill ME, Langaas M (2021) A new method for exploring gene–gene and gene–environment interactions in GWAS with tree ensemble methods and SHAP values. BMC Bioinformatics 22:230. doi: 10.1186/s12859-021-04041-7

3. Martini ML, Neifert SN, Shuman WH, Chapman EK, Schüpper AJ, Oermann EK, Mocco J, Todd M, Torner JC, Molyneux A, Mayer S, Roux PL, Vergouwen MDI, Rinkel GJE, Wong GKC, Kirkpatrick P, Quinn A, Hänggi D, Etminan N, Van Den Bergh WM, Jaja BNR, Cusimano M, Schweizer TA, Suarez JI, Fukuda H, Yamagata S, Lo B, Leonardo De Oliveira Manoel A, Boogaarts HD, Macdonald RL, __ (2022) Rescue therapy for vasospasm following aneurysmal subarachnoid hemorrhage: a propensity score–matched analysis with machine learning. J Neurosurg 136:134–147. doi: 10.3171/2020.12.JNS203778

4. Wu J, Tao G, Xie S, Yang H, Qi F, Bao N, Li Z, Chang G, Xiao H (2025) Prediction of three-

year all-cause mortality in patients with heart failure and atrial fibrillation using the CatBoost model. BMC Cardiovasc Disord 25:466. doi: 10.1186/s12872-025-04928-w

5. Zhang M, Shen T, Li Y, Li Q, Lou Y (2025) Exploring the complex associations between community public spaces and healthy aging: an explainable analysis using catboost and SHAP. BMC Public Health 25:2200. doi: 10.1186/s12889-025-23402-y
