# Supplementary material for: Machine Learning-Identified Potential Interaction Between Clazosentan and Nicardipine in Patients with Subarachnoid Hemorrhage
Source: J Clin Med. 2026 Feb 10;15(4):1383. doi: 10.3390/jcm15041383 (PMC12941893; doi:10.3390/jcm15041383)
Supplement: Supplementary file 1 [file jcm-15-01383-s001.zip › JCM_SupTable S1_251227infact.pdf]

Supplementary Table S1. Multivariable logistic regression analysis of risk factors for cerebral infarction (n = 544)

	Univariable analysis			Multivariable analysis	
	Cerebral infarction (-) (n = 422, 77.6%)	Cerebral infarction (+) (n = 122, 22.4%)	p values	OR (95% confidence interval)	p values
Age; 60 ≤ years (%)	263 (62.3%)	79 (64.8%)	0.624	1.12 (0.65–1.93)	0.676
Interaction between clazosentan and age				0.82 (0.32–2.11)	0.684
Sex (%female)	305 (72.3%)	84 (68.9%)	0.461	0.83 (0.52–1.33)	0.440
Past history					
Hypertension (%)	182 (43.1%)	42 (34.4%)	0.085		
Diabetes mellitus (%)	51 (12.1%)	9 (7.4%)	0.144		
Stroke (%)	22 (5.2%)	4 (3.3%)	0.375		
WFNS grade					
I (%)	190 (45.0%)	57 (46.7%)	0.841		
II (%)	94 (22.3%)	27 (22.1%)			
III (%)	20 (4.7%)	3 (2.5%)			
IV (%)	62 (14.7%)	17 (13.9%)			
V (%)	56 (13.3%)	18 (14.8%)			
Aneurysm size (mm), mean (SD)	5.8 (3.3)	6.4 (4.0)	0.108		
Aneurysm location			0.588		
ACA or ACoA (%)	125 (29.6%)	43 (35.2%)			
ICA (%)	146 (34.6%)	42 (34.4%)			
MCA (%)	101 (23.9%)	26 (21.4%)			
VA, BA, PCA, PICA (%)	50 (11.9%)	11 (9.0%)			
Fisher CT group					
1–3 (%)	318 (75.3%)	103 (84.5%)			
4 (%)	104 (24.7%)	19 (15.5%)	0.035*	0.54 (0.31–0.94)	0.030*
Surgical procedure					
Endovascular coiling, not surgical clipping (%endovascular coiling)	203 (48.1%)	59 (48.4%)	0.960		
Spinal drainage (%)	157 (37.2%)	39 (32.0%)	0.289		
Ventricular drainage (%)	94 (22.3%)	45 (36.9%)	0.001**	1.74 (1.02–2.99)	0.044*
Cisternal drainage (%)	64 (15.2%)	31 (25.4%)	0.009**	1.71 (0.80–3.63)	0.163

Interaction between clazosentan and cisternal drainage				0.35 (0.10–1.18)	0.090
Cerebral vasospasm prophylaxis and other medications					
Clazosentan (%)	152 (36.0%)	33 (27.0%)	0.065	0.44 (0.11–1.36)	0.155
Fasudil (%)	339 (80.3%)	106 (86.9%)	0.098	0.95 (0.40–2.26)	0.912
Cilostazol (%)	312 (73.9%)	93 (76.2%)	0.609		
Statin (%)	145 (34.4%)	57 (46.7%)	0.013*	1.11 (0.61–2.04)	0.734
Interaction between clazosentan and statin				2.51 (0.85–7.48)	0.097
Nicardipine (%)	71 (16.8%)	24 (19.7%)	0.466		
Antiepileptic drug (%)	161 (38.2%)	58 (47.5%)	0.063		
Complications and outcomes					
Angiographic vasospasm (%)	47 (11.1%)	65 (53.3%)	<0.001***		
Symptomatic vasospasm (%)	26 (6.2%)	62 (50.8%)	<0.001***		
IVR against cerebral vasospasm (%)	7 (1.6%)	16 (13.1%)	<0.001***		
Cerebral complication (%)	150 (35.5%)	65 (53.3%)	<0.001***		
Systemic complication (%)	190 (45.0%)	45 (36.9%)	0.110		
mRS score 0–2 at discharge (%)	235 (55.7%)	43 (35.2%)	<0.001***		
mRS score 0–2 at 6 months (%) (n = 406)	221/304 (72.7%)	48/102 (47.1%)	<0.001***		

There were no missing values. In this multivariable analysis, we included the items that were significant in the univariable analysis, along with the three items with the largest SHapley Additive exPlanations interaction values in the machine learning model.

Abbreviations: ACA; anterior cerebral artery, ACoA; anterior communicating artery, BA; basilar artery, Fasudil; fasudil hydrochloride hydrate, ICA; internal carotid artery, IVR; interventional radiology, MCA; middle cerebral artery, mRS; modified Rankin Scale, OR; odds ratio, PCA; posterior cerebral artery, PICA; posterior inferior cerebellar artery, VA; vertebral artery, WFNS; World Federation of Neurosurgical Societies, *; $p < 0.05$, **; $p < 0.01$, ***; $p < 0.001$.
