# Supplementary material for: Machine Learning-Identified Potential Interaction Between Clazosentan and Nicardipine in Patients with Subarachnoid Hemorrhage
Source: J Clin Med. 2026 Feb 10;15(4):1383. doi: 10.3390/jcm15041383 (PMC12941893; doi:10.3390/jcm15041383)
Supplement: Supplementary file 1 [file jcm-15-01383-s001.zip › JCM_SupTable S2_251227mRSdischarge.pdf]

Supplementary Table S2. Multivariable logistic regression analysis of risk factors for poor outcomes (mRS score ≥ 3) at discharge (n = 544)

	Univariable analysis			Multivariable analysis	
	mRS score 0–2 at discharge (n = 278, 51.1%)	mRS score 3–6 at discharge (n = 266, 48.9%)	p values	OR (95% confidence interval)	p values
Age; 60 ≤ years (%)	174 (62.6%)	228 (85.7%)	<0.001***	3.56 (2.02–6.26)	<0.001***
Interaction between clazosentan and age				0.86 (0.33–2.28)	0.756
Sex (%female)	201 (72.3%)	188 (70.7%)	0.675	0.78 (0.49–1.25)	0.304
Past history					
Hypertension (%)	101 (36.3%)	123 (46.2%)	0.019*	1.72 (1.04–2.87)	0.036*
Interaction between clazosentan and hypertension				0.88 (0.35–2.23)	0.787
Diabetes mellitus (%)	25 (9.0%)	35 (13.2%)	0.121		
Stroke (%)	9 (3.2%)	17 (6.4%)	0.087		
WFNS grade					
I (%)	140 (50.4%)	107 (40.2%)	<0.001***	Reference	
II (%)	81 (29.1%)	40 (15.0%)		0.73 (0.43–1.25)	0.260
III (%)	12 (4.3%)	11 (4.1%)		1.01 (0.37–2.75)	0.980
IV (%)	28 (10.1%)	51 (19.2%)		2.64 (1.42–4.93)	0.002**
V (%)	17 (6.1%)	57 (21.4%)		3.35 (1.62–6.96)	0.001**
Aneurysm size (mm), mean (SD)	5.3 (2.6)	6.6 (4.1)	<0.001***	1.11 (1.03–1.19)	0.007**
Aneurysm location			0.735		
ACA or ACoA (%)	83 (29.9%)	85 (32.0%)			
ICA (%)	102 (36.7%)	86 (32.3%)			
MCA (%)	64 (23.0%)	63 (23.7%)			
VA, BA, PCA, PICA (%)	29 (10.4%)	32 (12.0%)			
Fisher CT group					
1–3 (%)	246 (88.5%)	175 (65.8%)			
4 (%)	32 (11.5%)	91 (34.2%)	<0.001***	2.63 (1.42–4.91)	0.002**
Interaction between clazosentan and Fisher group 4				2.55 (0.75–8.76)	0.136

Surgical procedure					
Endovascular coiling, not surgical clipping (%endovascular coiling)	144 (51.8%)	118 (44.4%)	0.083		
Spinal drainage (%)	102 (36.7%)	94 (35.3%)	0.743		
Ventricular drainage (%)	47 (16.9%)	92 (34.6%)	<0.001***	2.84 (1.61–5.01)	<0.001***
Cisternal drainage (%)	38 (13.7%)	57 (21.4%)	0.017*	1.39 (0.72–2.70)	0.331
Cerebral vasospasm prophylaxis and other medications					
Clazosentan (%)	115 (41.4%)	70 (26.3%)	<0.001***	0.80 (0.31–2.06)	0.648
Fasudil (%)	216 (77.7%)	229 (86.1%)	0.011*	0.99 (0.45–2.15)	0.971
Cilostazol (%)	216 (77.7%)	189 (71.1%)	0.076		
Statin (%)	98 (35.3%)	104 (39.1%)	0.353		
Nicardipine (%)	47 (16.9%)	48 (18.0%)	0.727		
Antiepileptic drug (%)	115 (41.4%)	104 (39.1%)	0.590		
Complications and outcomes					
Angiographic vasospasm (%)	41 (14.7%)	71 (26.7%)	<0.001***	†	
Symptomatic vasospasm (%)	18 (6.5%)	70 (26.3%)	<0.001***	3.57 (1.77–7.22)	<0.001***
IVR against cerebral vasospasm (%)	9 (3.2%)	14 (5.3%)	0.289		
Cerebral infarction (%)	43 (15.5%)	79 (29.7%)	<0.001***	1.47 (0.81–2.66)	0.205
Cerebral complication (%)	84 (30.2%)	131 (49.2%)	<0.001***	1.44 (0.89–2.31)	0.131
Systemic complication (%)	98 (35.3%)	137 (51.5%)	<0.001***	1.30 (0.82–2.04)	0.265
mRS score 0–2 at 6 months (%) (n = 406)	215/223 (96.4%)	54/183 (29.5%)	<0.001***		

There were no missing values. In this multivariable analysis, we included the items that were significant in the univariable analysis, along with the three items with the largest SHapley Additive exPlanations interaction values in the machine learning model.

Abbreviations: ACA; anterior cerebral artery, ACoA; anterior communicating artery, BA; basilar artery, Fasudil; fasudil hydrochloride hydrate, ICA; internal carotid artery, IVR; interventional radiology, MCA; middle cerebral artery, mRS; modified Rankin Scale, OR; odds ratio, PCA; posterior cerebral artery, PICA; posterior inferior cerebellar artery, VA; vertebral artery, WFNS; World Federation of Neurosurgical Societies, *; $p < 0.05$, **; $p < 0.01$, ***; $p < 0.001$.

†; Presence of angiographic and symptomatic vasospasm has strong correlation ($r = 0.862$, $p < 0.001$ by Spearman's correlation coefficient). Therefore, to avoid multicollinearity, only symptomatic vasospasm was included in the multivariable analysis.
