# Supplementary material for: Machine Learning-Identified Potential Interaction Between Clazosentan and Nicardipine in Patients with Subarachnoid Hemorrhage
Source: J Clin Med. 2026 Feb 10;15(4):1383. doi: 10.3390/jcm15041383 (PMC12941893; doi:10.3390/jcm15041383)
Supplement: Supplementary file 1 [file jcm-15-01383-s001.zip › JCM_SupTable S3_251227mRSatdischarge.pdf]

Supplementary Table S3. Multivariable logistic regression analysis of risk factors for poor outcomes (mRS score ≥ 3) at 6 months (n = 406)

	Univariable analysis			Multivariable analysis	
	mRS score 0–2 at 6 months (n = 269, 66.3%)	mRS score 3–6 at 6 months (n = 137, 33.7%)	p values	OR (lower-upper confidence interval)	p values
Age; 60 ≤ years (%)	170 (63.2%)	118 (86.1%)	<0.001***	3.29 (1.62–6.65)	<0.001***
Sex (%female)	194 (72.1%)	99 (72.3%)	0.976	0.82 (0.44–1.51)	0.468
Past history					
Hypertension (%)	95 (35.3%)	63 (46.0%)	0.037*	1.78 (1.06–3.34)	0.032*
Diabetes mellitus (%)	35 (13.0%)	16 (11.7%)	0.702		
Stroke (%)	5 (1.9%)	8 (5.8%)	0.032*	1.91 (0.39–9.18)	0.417
WFNS grade					
I (%)	131 (48.7%)	60 (43.8%)	<0.001***	Reference	
II (%)	85 (31.6%)	16 (11.7%)		0.27 (0.07–1.09)	0.260
III (%)	14 (5.2%)	5 (3.6%)		0.35 (0.09–1.28)	0.192
IV (%)	27 (10.0%)	28 (20.4%)		1.74 (1.01–2.98)	0.002**
V (%)	12 (4.5%)	28 (20.4%)		2.32 (1.04–5.16)	0.001**
Interaction between clazosentan and WFNS grade I				Reference	
Interaction between clazosentan and WFNS grade II				9.92 (1.88–52.35)	0.007**
Interaction between clazosentan and WFNS grade III				0.70 (0.05–9.45)	0.792
Interaction between clazosentan and WFNS grade IV				9.61 (1.62–57.01)	0.013*
Interaction between clazosentan and WFNS grade V				8.28 (1.00–65.82)	0.049*
Aneurysm size (mm), mean (SD)	5.5 (2.5)	6.9 (4.7)	<0.001***	1.15 (1.03–1.29)	0.014*
Interaction between clazosentan and aneurysm size				1.01 (0.80–1.27)	0.913
Aneurysm location			0.479		
ACA or ACoA (%)	78 (29.0%)	48 (35.0%)			

ICA (%)	100 (37.2%)	41 (29.9%)			
MCA (%)	63 (23.4%)	33 (24.1%)			
VA, BA, PCA, PICA (%)	28 (10.4%)	15 (11.0%)			
Fisher CT group					
1–3 (%)	237 (88.1%)	99 (72.3%)			
4 (not 1–3) (%)	32 (11.9%)	38 (27.7%)	<0.001***	2.01 (1.00–4.20)	0.049*
Surgical procedure					
Endovascular coiling, not surgical clipping (%endovascular coiling)	131 (48.7%)	58 (42.3%)	0.224		
Spinal drainage (%)	125 (46.5%)	51 (37.2%)	0.076		
Ventricular drainage (%)	54 (20.1%)	58 (42.3%)	<0.001***	3.43 (1.68–6.98)	0.001**
Interaction between clazosentan and ventricular drainage				2.69 (0.71–10.30)	0.147
Cisternal drainage (%)	49 (18.2%)	36 (26.3%)	0.059		
Cerebral vasospasm prophylaxis and other medications					
Clazosentan (%)	125 (46.5%)	34 (24.8%)	<0.001***	0.08 (0.01–0.66)	0.019*
Fasudil (%)	201 (74.7%)	116 (84.7%)	0.022*	0.50 (0.17–1.50)	0.216
Cilostazol (%)	194 (72.1%)	96 (70.1%)	0.666		
Statin (%)	116 (43.1%)	66 (48.2%)	0.330		
Nicardipine (%)	55 (20.4%)	25 (18.2%)	0.599		
Antiepileptic drug (%)	127 (47.2%)	54 (39.4%)	0.135		
Complications					
Angiographic vasospasm (%)	41 (15.2%)	43 (31.4%)	<0.001***	†	
Symptomatic vasospasm (%)	24 (8.9%)	43 (31.4%)	<0.001***	2.23 (1.09–4.88)	0.045*
IVR against cerebral vasospasm (%)	12 (4.5%)	9 (6.6%)	0.355		
Cerebral infarction (%)	48 (17.8%)	54 (39.4%)	<0.001***	1.73 (0.86–3.66)	0.119
Cerebral complication (%)	49 (18.2%)	60 (43.8%)	<0.001***	3.07 (1.63–5.78)	<0.001***
Systemic complication (%)	71 (26.4%)	71 (51.8%)	<0.001***	2.47 (1.39–4.38)	0.002**

There were no missing values. In this multivariable analysis, we included the items that were significant in the univariable analysis, along with the three items with the largest SHapley Additive exPlanations interaction values in the machine learning model.

Abbreviations: ACA; anterior cerebral artery, ACoA; anterior communicating artery, BA; basilar artery,

Fasudil; fasudil hydrochloride hydrate, ICA; internal carotid artery, IVR; interventional radiology, MCA; middle cerebral artery, mRS; modified Rankin Scale, OR; odds ratio, PCA; posterior cerebral artery, PICA; posterior inferior cerebellar artery, VA; vertebral artery, WFNS; World Federation of Neurosurgical Societies, *; $p < 0.05$, **; $p < 0.01$, ***; $p < 0.001$.

†; The presence of angiographic and symptomatic vasospasm has a strong correlation ($r = 0.862$, $p < 0.001$ by Spearman's correlation coefficient). Therefore, to avoid multicollinearity, only symptomatic vasospasm was included in the multivariable analysis.
