# Supplementary material for: Machine Learning-Identified Potential Interaction Between Clazosentan and Nicardipine in Patients with Subarachnoid Hemorrhage
Source: J Clin Med. 2026 Feb 10;15(4):1383. doi: 10.3390/jcm15041383 (PMC12941893; doi:10.3390/jcm15041383)
Supplement: Supplementary file 1 [file jcm-15-01383-s001.zip › JCM_SupTable S4 0119strata.pdf]

Supplementary Table S4. Multivariable logistic regression–derived adjusted predicted probabilities for AVS, SVS, cerebral infarction, and mRS outcomes according to clazosentan and nicardipine use

Outcome: AVS (n = 544) *	Adjusted predicted probability (Wald 95% confidence interval)
Clazosentan (+) / Nicardipine (+)	0.43 (0.12–0.80)
Clazosentan (+) / Nicardipine (−)	0.10 (0.06–0.16)
Clazosentan (−) / Nicardipine (+)	0.07 (0.04–0.15)
Clazosentan (−) / Nicardipine (−)	0.21 (0.15–0.30)
Outcome: SVS (n = 544) **	
Clazosentan (+) / Nicardipine (+)	0.21 (0.03–0.71)
Clazosentan (+) / Nicardipine (−)	0.13 (0.08–0.20)
Clazosentan (−) / Nicardipine (+)	0.19 (0.08–0.38)
Clazosentan (−) / Nicardipine (−)	0.35 (0.22–0.51)
Outcome: cerebral infarction (n = 544) ***	
Clazosentan (+) / Nicardipine (+)	0.46 (0.13–0.83)
Clazosentan (+) / Nicardipine (−)	0.19 (0.13–0.26)
Clazosentan (−) / Nicardipine (+)	0.39 (0.23–0.58)
Clazosentan (−) / Nicardipine (−)	0.31 (0.20–0.43)
Outcome: mRS ≥3 at discharge (n = 544) †	
Clazosentan (+) / Nicardipine (+)	0.69 (0.19–0.96)
Clazosentan (+) / Nicardipine (−)	0.29 (0.19–0.40)
Clazosentan (−) / Nicardipine (+)	0.22 (0.11–0.39)
Clazosentan (−) / Nicardipine (−)	0.26 (0.16–0.38)
Outcome: mRS ≥3 at 6 months (n = 406) ‡	
Clazosentan (+) / Nicardipine (+)	0.27 (0.03–0.83)
Clazosentan (+) / Nicardipine (−)	0.44 (0.31–0.58)
Clazosentan (−) / Nicardipine (+)	0.60 (0.36–0.80)
Clazosentan (−) / Nicardipine (−)	0.70 (0.53–0.82)

Adjusted predicted probabilities were estimated using the same covariates as those included in the multivariable model. There were no missing values. Planned pairwise comparisons were pre-specified as follows: (i) within Nicardipine (−), Clazosentan (+) vs (−); (ii) within Clazosentan (−), Nicardipine (+) vs (−); and (iii) within Nicardipine (+), Clazosentan (+) vs (−). Pairwise comparisons (pre-specified) are reported below; p values are unadjusted.

*: Pairwise comparisons of adjusted predicted probabilities for AVS (Wald 95% CI) showed significant differences for Clazosentan(−)/Nicardipine(−) vs Clazosentan(+)/Nicardipine(−) (difference = 0.11, 95% CI 0.05–0.18; $p < 0.001$) and for Clazosentan(−)/Nicardipine(−) vs Clazosentan(−)/Nicardipine(+) (difference = 0.14, 95% CI 0.06–0.21; $p < 0.001$). In contrast, the combined-exposure stratum (Clazosentan(+)/Nicardipine(+)) did not differ significantly from the other strata in pairwise comparisons (all $p > 0.05$), likely reflecting the small sample size in this stratum.

**: Pairwise comparisons of adjusted predicted probabilities for SVS (Wald 95% CI) showed significant differences for Clazosentan(−)/Nicardipine(−) vs Clazosentan(+)/Nicardipine(−) (difference = 0.22, 95% CI 0.06–0.38; $p = 0.007$) and for Clazosentan(−)/Nicardipine(−) vs Clazosentan(−)/Nicardipine(+) (difference = 0.15, 95% CI 0.02–0.29; $p = 0.024$).

***: Pairwise comparisons of adjusted predicted probabilities for cerebral infarction (Wald 95% CI) identified a significant difference between Clazosentan(−)/Nicardipine(+) and Clazosentan(+)/Nicardipine(+) (difference = 0.20, 95% CI 0.02–0.39; $p = 0.033$).

†: Pairwise comparisons of adjusted predicted probabilities for poor outcome (mRS ≥3) at discharge (Wald 95% CI) showed no statistically significant differences in the pre-specified pairwise comparisons (all $p > 0.05$).

‡: Pairwise comparisons of adjusted predicted probabilities for poor outcome (mRS ≥3) at 6 months (Wald 95% CI) showed a significant difference between Clazosentan(−)/Nicardipine(−) and Clazosentan(+)/Nicardipine(−) (difference = 0.26, 95% CI 0.08–0.44; $p = 0.005$).

Abbreviations: AVS; angiographic vasospasm, mRS; modified Rankin Scale, SVS; symptomatic vasospasm
