# Supplementary material for: Machine Learning-Identified Potential Interaction Between Clazosentan and Nicardipine in Patients with Subarachnoid Hemorrhage
Source: J Clin Med. 2026 Feb 10;15(4):1383. doi: 10.3390/jcm15041383 (PMC12941893; doi:10.3390/jcm15041383)
Supplement: Supplementary file 1 [file jcm-15-01383-s001.zip › JCM_SupTable S5_20260119_AVS timevariable.pdf]

Supplementary Table S5 Multivariable logistic regression analysis of risk factors for angiographic vasospasm (sensitivity analysis) (n = 544)

	Univariable analysis			Multivariable analysis	
	Angiographic vasospasm (-) (n = 432, 79.4%)	Angiographic vasospasm (+) (n = 112, 20.6%)	p values	OR (95% confidence interval)	p values
Post-April 2022 (%)	245 (56.7%)	60 (53.6%)	0.551	1.21 (0.71–2.04)	0.480
Age; 60 ≤ years (%)	272 (63.0%)	70 (62.5%)	0.688	0.86 (0.50–1.49)	0.594
Interaction between clazosentan and age				1.51 (0.55–4.19)	0.423
Sex (%female)	307 (71.1%)	82 (73.2%)	0.653	1.13 (0.69–1.85)	0.630
Past history					
Hypertension (%)	189 (43.8%)	35 (31.3%)	0.017*	0.48 (0.28–0.82)	0.007**
Interaction between clazosentan and hypertension				1.44 (0.49–4.26)	0.512
Diabetes mellitus (%)	46 (10.6%)	14 (12.5%)	0.577		
Stroke (%)	22 (5.1%)	4 (3.6%)	0.498		
WFNS grade					
I (%)	202 (46.8%)	45 (40.2%)	0.185		
II (%)	98 (22.7%)	23 (20.5%)			
III (%)	20 (4.6%)	3 (2.7%)			
IV (%)	60 (13.9%)	19 (17.0%)			
V (%)	52 (12.0%)	22 (19.6%)			
Aneurysm size (mm), mean (SD)	5.9 (3.5)	6.1 (3.4)	0.349		
Aneurysm location			0.275		
ACA or ACoA (%)	133 (30.8%)	35 (31.3%)			
ICA (%)	148 (34.2%)	40 (35.7%)			
MCA (%)	97 (22.5%)	30 (26.8%)			
VA, BA, PCA, PICA (%)	54 (12.5%)	7 (6.2%)			
Fisher CT group					
1−3 (%)	342 (79.2%)	79 (70.5%)			
4 (%)	90 (20.8%)	33 (29.5%)	0.052		
Surgical procedure					
Endovascular coiling, not surgical clipping (%endovascular coiling)	210 (48.6%)	52 (46.4%)	0.680		
Spinal drainage (%)	147 (34.0%)	49 (43.8%)	0.056		
Ventricular drainage (%)	105 (24.3%)	34 (30.4%)	0.191		
Cisternal drainage (%)	67 (15.5%)	28 (25.0%)	0.018*	1.83 (1.06–3.15)	0.029*

Cerebral vasospasm prophylaxis and other medications					
Clazosentan (%)	157 (36.3%)	28 (25.0%)	0.024*	0.24 (0.09–0.65)	0.005**
Fasudil (%)	348 (80.6%)	97 (86.6%)	0.139	0.88 (0.37–2.09)	0.777
Cilostazol (%)	330 (76.4%)	75 (67.0%)	0.042*	0.69 (0.37–1.11)	0.129
Statin (%)	160 (37.0%)	42 (37.5%)	0.928		
Nicardipine (%)	82 (19.0%)	13 (11.6%)	0.067	0.39 (0.19–0.80)	0.011*
Interaction between clazosentan and nicardipine				1.86 (1.41–2.93)	0.002**
Antiepileptic drug (%)	178 (41.2%)	41 (36.6%)	0.377		
Outcomes and complications					
mRS score 0–2 at discharge (%)	237 (54.9%)	41 (36.6%)	<0.001***		
mRS score 0–2 at 6 months (%) (n = 406)	228/322 (70.8%)	41/84 (48.8%)	0.002**		
Symptomatic vasospasm (%)	0 (0%)	87 (77.7%)	<0.001***		
IVR against cerebral vasospasm (%)	3 (0.7%)	20 (17.9%)	<0.001***		
Cerebral infarction (%)	57 (13.2%)	65 (58.0%)	<0.001***		
Cerebral complication (%)	153 (35.4%)	62 (55.4%)	<0.001***		
Systemic complication (%)	177 (41.0%)	58 (51.8%)	0.040*		

As a sensitivity analysis, we additionally included a binary calendar-time variable (post–April 2022 period) in the multivariable models to account for potential time-related practice changes. There were no missing values. In this multivariable analysis, we included the items that were significant in the univariable analysis, along with the three items with the largest SHapley Additive exPlanations interaction values in the machine learning model.

Abbreviations: ACA; anterior cerebral artery, ACoA; anterior communicating artery, BA; basilar artery, Fasudil; fasudil hydrochloride hydrate, ICA; internal carotid artery, IVR; interventional radiology, MCA; middle cerebral artery, mRS; modified Rankin Scale, OR; odds ratio, PCA; posterior cerebral artery, PICA; posterior inferior cerebellar artery, VA; vertebral artery, WFNS; World Federation of Neurosurgical Societies, *; $p < 0.05$, **; $p < 0.01$, ***; $p < 0.001$.
