# Supplementary material for: Machine Learning-Identified Potential Interaction Between Clazosentan and Nicardipine in Patients with Subarachnoid Hemorrhage
Source: J Clin Med. 2026 Feb 10;15(4):1383. doi: 10.3390/jcm15041383 (PMC12941893; doi:10.3390/jcm15041383)
Supplement: Supplementary file 1 [file jcm-15-01383-s001.zip › JCM_SupTable S6_20260119_SVS timevariable.pdf]

Supplementary Table S6 Multivariable logistic regression analysis of risk factors for symptomatic vasospasm (sensitivity analysis) (n = 544)

	Univariable analysis			Multivariable analysis	
	Symptomatic vasospasm (-) (n = 457, 84.0%)	Symptomatic vasospasm (+) (n = 87, 16.0%)	p values	OR (95% confidence interval)	p values
Post-April 2022 (%)	260 (57.0%)	45 (51.1%)	0.309	1.07 (0.61–1.87)	0.824
Age; 60 ≤ years (%)	283 (61.9%)	59 (67.8%)	0.928	1.01 (0.60–1.83)	0.965
Interaction between clazosentan and age				1.56 (0.46–5.22)	0.473
Sex (%female)	325 (71.1%)	64 (73.6%)	0.782	1.03 (0.60–1.76)	0.473
Past history					
Hypertension (%)	196 (42.9%)	28 (32.2%)	0.051		
Diabetes mellitus (%)	49 (10.7%)	11 (12.5%)	0.631		
Stroke (%)	22 (4.8%)	4 (4.5%)	0.907		
WFNS grade					
I (%)	212 (46.4%)	35 (39.8%)	0.089		
II (%)	106 (23.2%)	15 (17.0%)			
III (%)	20 (4.4%)	3 (3.4%)			
IV (%)	63 (13.8%)	16 (18.2%)			
V (%)	55 (12.1%)	19 (21.6%)			
Aneurysm size (mm), mean (SD)	5.8 (3.5)	6.4 (3.6)	0.105	1.02 (0.96–1.09)	0.565
Interaction between clazosentan and aneurysm size				1.07 (0.89–1.28)	0.466
Aneurysm location			0.318		
ACA or ACoA (%)	144 (31.5%)	24 (27.6%)			
ICA (%)	156 (34.2%)	32 (36.8%)			
MCA (%)	102 (22.3%)	25 (28.7%)			
VA, BA, PCA, PICA (%)	55 (12.0%)	6 (6.9%)			
Fisher CT group					
1–3 (%)	358 (78.3%)	63 (72.4%)			
4 (%)	99 (21.7%)	24 (27.6%)	0.253		
Surgical procedure					
Endovascular coiling, not surgical clipping (%endovascular coiling)	222 (48.6%)	40 (46.0%)	0.579		
Spinal drainage (%)	159 (34.9%)	37 (42.5%)	0.199		
Ventricular drainage (%)	107 (23.5%)	32 (36.8%)	0.011*	1.28 (0.70–2.32)	0.421
Cisternal drainage (%)	71 (15.6%)	24 (27.6%)	0.008**	1.83 (0.94–3.56)	0.076

Cerebral vasospasm prophylaxis and other medications					
Clazosentan (%)	166 (36.4%)	19 (21.6%)	0.007**	0.17 (0.04–0.79)	0.024*
Fasudil (%)	369 (80.7%)	76 (87.4%)	0.226	0.58 (0.20–1.64)	0.302
Cilostazol (%)	346 (75.7%)	59 (67.8%)	0.082	0.73 (0.37–1.23)	0.236
Statin (%)	168 (36.8%)	34 (39.1%)	0.750		
Nicardipine (%)	85 (18.6%)	10 (11.4%)	0.001**	0.47 (0.22–1.01)	0.051
Interaction between clazosentan and nicardipine				4.43 (0.39–48.1)	0.226
Antiepileptic drug (%)	189 (41.4%)	30 (34.5%)	0.198		
Outcomes and complications (%)					
mRS score 0–2 at discharge (%)	260 (56.9%)	18 (20.5%)	<0.001***		
mRS score 0–2 at 6 months (%) (n = 406)	245/339 (72.3%)	43/67 (64.2%)	<0.001***		
Angiographic vasospasm (%)	24 (5.3%)	87 (100%)	<0.001***		
IVR against cerebral vasospasm (%)	3 (0.7%)	20 (23.0%)	<0.001***		
Cerebral infarction (%)	60 (13.2%)	62 (71.3%)	<0.001***		
Cerebral complication (%)	164 (36.0%)	51 (58.6%)	<0.001***		
Systemic complication (%)	190 (41.6%)	45 (51.7%)	0.101		

As a sensitivity analysis, we additionally included a binary calendar-time variable (post–April 2022 period) in the multivariable models to account for potential time-related practice changes. There were no missing values. In this multivariable analysis, we included the items that were significant in the univariable analysis, along with the three items with the largest SHapley Additive exPlanations interaction values in the machine learning model.

Abbreviations: ACA; anterior cerebral artery, ACoA; anterior communicating artery, BA; basilar artery, Fasudil; fasudil hydrochloride hydrate, ICA; internal carotid artery, IVR; interventional radiology, MCA; middle cerebral artery, mRS; modified Rankin Scale, OR; odds ratio, PCA; posterior cerebral artery, PICA; posterior inferior cerebellar artery, VA; vertebral artery, WFNS; World Federation of Neurosurgical Societies, *; p < 0.05, **; p < 0.01, ***; p < 0.001.
