# Supplementary material for: Machine Learning-Identified Potential Interaction Between Clazosentan and Nicardipine in Patients with Subarachnoid Hemorrhage
Source: J Clin Med. 2026 Feb 10;15(4):1383. doi: 10.3390/jcm15041383 (PMC12941893; doi:10.3390/jcm15041383)
Supplement: Supplementary file 1 [file jcm-15-01383-s001.zip › JCM_SupTable S7_260119infarct_timevariable.pdf]

Supplementary Table S7. Multivariable logistic regression analysis of risk factors for cerebral infarction (sensitivity analysis) (n = 544)

	Univariable analysis			Multivariable analysis	
	Cerebral infarction (-) (n = 422, 77.6%)	Cerebral infarction (+) (n = 122, 22.4%)	p values	OR (95% confidence interval)	p values
Post-April 2022 (%)	241 (57.1%)	64 (52.5%)	0.362	1.15 (0.69–1.92)	0.601
Age; 60 ≤ years (%)	263 (62.3%)	79 (64.8%)	0.624	1.10 (0.64–1.90)	0.721
Interaction between clazosentan and age				0.90 (0.35–2.36)	0.841
Sex (%female)	305 (72.3%)	84 (68.9%)	0.461	0.83 (0.52–1.32)	0.430
Past history					
Hypertension (%)	182 (43.1%)	42 (34.4%)	0.085		
Diabetes mellitus (%)	51 (12.1%)	9 (7.4%)	0.144		
Stroke (%)	22 (5.2%)	4 (3.3%)	0.375		
WFNS grade					
I (%)	190 (45.0%)	57 (46.7%)	0.841		
II (%)	94 (22.3%)	27 (22.1%)			
III (%)	20 (4.7%)	3 (2.5%)			
IV (%)	62 (14.7%)	17 (13.9%)			
V (%)	56 (13.3%)	18 (14.8%)			
Aneurysm size (mm), mean (SD)	5.79 (3.30)	6.42 (4.04)	0.108		
Aneurysm location			0.588		
ACA or ACoA (%)	125 (29.6%)	43 (35.2%)			
ICA (%)	146 (34.6%)	42 (34.4%)			
MCA (%)	101 (23.9%)	26 (21.4%)			
VA, BA, PCA, PICA (%)	50 (11.9%)	11 (9.0%)			
Fisher CT group					
1−3 (%)	318 (75.3%)	103 (84.5%)			
4 (%)	104 (24.7%)	19 (15.5%)	0.035*	0.54 (0.31–0.94)	0.029*
Surgical procedure					
Endovascular coiling, not surgical clipping (%endovascular coiling)	203 (48.1%)	59 (48.4%)	0.960		
Spinal drainage (%)	157 (37.2%)	39 (32.0%)	0.289		
Ventricular drainage (%)	94 (22.3%)	45 (36.9%)	0.001**	1.68 (0.97–2.89)	0.061

Cisternal drainage (%)	64 (15.2%)	31 (25.4%)	0.009**	1.71 (0.80–3.63)	0.164
Interaction between clazosentan and cisternal drainage				0.37 (0.11–1.24)	0.107
Cerebral vasospasm prophylaxis and other medications					
Clazosentan (%)	152 (36.0%)	33 (27.0%)	0.065	0.46 (0.14–1.51)	0.164
Fasudil (%)	339 (80.3%)	106 (86.9%)	0.098	0.91 (0.42–2.43)	0.966
Cilostazol (%)	312 (73.9%)	93 (76.2%)	0.609		
Statin (%)	145 (34.4%)	57 (46.7%)	0.013*	1.17 (0.62–2.18)	0.638
Interaction between clazosentan and statin				2.48 (0.82–7.35)	0.108
Nicardipine (%)	71 (16.8%)	24 (19.7%)	0.466		
Antiepileptic drug (%)	161 (38.2%)	58 (47.5%)	0.063		
Complications and outcomes					
Angiographic vasospasm (%)	47 (11.1%)	65 (53.3%)	<0.001***		
Symptomatic vasospasm (%)	26 (6.2%)	62 (50.8%)	<0.001***		
IVR against cerebral vasospasm (%)	7 (1.6%)	16 (13.1%)	<0.001***		
Cerebral complication (%)	150 (35.5%)	65 (53.3%)	<0.001***		
Systemic complication (%)	190 (45.0%)	45 (36.9%)	0.110		
mRS score 0–2 at discharge (%)	235 (55.7%)	43 (35.2%)	<0.001***		
mRS score 0–2 at 6 months (%) (n = 406)	221/304 (72.7%)	48/102 (47.1%)	<0.001***		

As a sensitivity analysis, we additionally included a binary calendar-time variable (post–April 2022 period) in the multivariable models to account for potential time-related practice changes. There were no missing values. In this multivariable analysis, we included the items that were significant in the univariable analysis, along with the three items with the largest SHapley Additive exPlanations interaction values in the machine learning model.

Abbreviations: ACA; anterior cerebral artery, ACoA; anterior communicating artery, BA; basilar artery, Fasudil; fasudil hydrochloride hydrate, ICA; internal carotid artery, IVR; interventional radiology, MCA; middle cerebral artery, mRS; modified Rankin Scale, OR; odds ratio, PCA; posterior cerebral artery, PICA; posterior inferior cerebellar artery, VA; vertebral artery, WFNS; World Federation of Neurosurgical Societies, *; $p < 0.05$, **; $p < 0.01$, ***; $p < 0.001$.
