# Supplementary material for: Machine Learning-Identified Potential Interaction Between Clazosentan and Nicardipine in Patients with Subarachnoid Hemorrhage
Source: J Clin Med. 2026 Feb 10;15(4):1383. doi: 10.3390/jcm15041383 (PMC12941893; doi:10.3390/jcm15041383)
Supplement: Supplementary file 1 [file jcm-15-01383-s001.zip › JCM_SupTable S8_260119mRSdischarge_timevariable.pdf]

Supplementary Table S8. Multivariable logistic regression analysis of risk factors for poor outcomes (mRS score ≥ 3) at discharge (sensitivity analysis) (n = 544)

	Univariable analysis			Multivariable analysis	
	mRS score 0–2 at discharge (n = 278, 51.1%)	mRS score 3–6 at discharge (n = 266, 48.9%)	p values	OR (95% confidence interval)	p values
Post-April 2022 (%)	151 (54.3%)	154 (57.9%)	0.401	1.90 (0.99–3.60)	0.113
Age; 60 ≤ years (%)	174 (62.6%)	228 (85.7%)	<0.001***	3.45 (1.95–6.09)	<0.001***
Interaction between clazosentan and age				0.88 (0.34–2.28)	0.792
Sex (%female)	201 (72.3%)	188 (70.7%)	0.675	0.78 (0.49–1.26)	0.311
Past history					
Hypertension (%)	101 (36.3%)	123 (46.2%)	0.019*	1.67 (1.00–2.79)	0.050*
Interaction between clazosentan and hypertension				0.86 (0.34–2.19)	0.755
Diabetes mellitus (%)	25 (9.0%)	35 (13.2%)	0.121		
Stroke (%)	9 (3.2%)	17 (6.4%)	0.087		
WFNS grade					
I (%)	140 (50.4%)	107 (40.2%)	<0.001***	Reference	
II (%)	81 (29.1%)	40 (15.0%)		0.78 (0.46–1.33)	0.357
III (%)	12 (4.3%)	11 (4.1%)		1.23 (0.45–3.38)	0.692
IV (%)	28 (10.1%)	51 (19.2%)		2.86 (1.52–5.39)	0.005**
V (%)	17 (6.1%)	57 (21.4%)		3.70 (1.77–7.74)	0.001**
Aneurysm size (mm), mean (SD)	5.3 (2.6)	6.6 (4.1)	<0.001***	1.10 (1.03–1.19)	0.008**
Aneurysm location			0.735		
ACA or ACoA (%)	83 (29.9%)	85 (32.0%)			
ICA (%)	102 (36.7%)	86 (32.3%)			
MCA (%)	64 (23.0%)	63 (23.7%)			
VA, BA, PCA, PICA (%)	29 (10.4%)	32 (12.0%)			
Fisher CT group					
1–3 (%)	246 (88.5%)	175 (65.8%)			
4 (%)	32 (11.5%)	91 (34.2%)	<0.001***	2.34 (1.25–4.40)	0.008*
Interaction between clazosentan				2.70 (0.79–9.23)	0.113

and Fisher group 4					
Surgical procedure					
Endovascular coiling, not surgical clipping (%endovascular coiling)	144 (51.8%)	118 (44.4%)	0.083		
Spinal drainage (%)	102 (36.7%)	94 (35.3%)	0.743		
Ventricular drainage (%)	47 (16.9%)	92 (34.6%)	<0.001***	2.72 (1.53–4.84)	<0.001***
Cisternal drainage (%)	38 (13.7%)	57 (21.4%)	0.017*	1.43 (0.73–2.79)	0.294
Cerebral vasospasm prophylaxis and other medications					
Clazosentan (%)	115 (41.4%)	70 (26.3%)	<0.001***	0.53 (0.20–1.45)	0.217
Fasudil (%)	216 (77.7%)	229 (86.1%)	0.011*	1.01 (0.46–2.19)	0.990
Cilostazol (%)	216 (77.7%)	189 (71.1%)	0.076		
Statin (%)	98 (35.3%)	104 (39.1%)	0.353		
Nicardipine (%)	47 (16.9%)	48 (18.0%)	0.727		
Antiepileptic drug (%)	115 (41.4%)	104 (39.1%)	0.590		
Complications and outcomes					
Angiographic vasospasm (%)	41 (14.7%)	71 (26.7%)	<0.001***	†	
Symptomatic vasospasm (%)	18 (6.5%)	70 (26.3%)	<0.001***	3.65 (1.79–7.43)	<0.001***
IVR against cerebral vasospasm (%)	9 (3.2%)	14 (5.3%)	0.289		
Cerebral infarction (%)	43 (15.5%)	79 (29.7%)	<0.001***	1.46 (0.80–2.65)	0.216
Cerebral complication (%)	84 (30.2%)	131 (49.2%)	<0.001***	1.44 (0.90–2.32)	0.132
Systemic complication (%)	98 (35.3%)	137 (51.5%)	<0.001***	1.30 (0.82–2.06)	0.260
mRS score 0–2 at 6 months (%) (n = 406)	215/223 (96.4%)	54/183 (29.5%)	<0.001***		

As a sensitivity analysis, we additionally included a binary calendar-time variable (post–April 2022 period) in the multivariable models to account for potential time-related practice changes. There were no missing values. In this multivariable analysis, we included the items that were significant in the univariable analysis, along with the three items with the largest SHapley Additive exPlanations interaction values in the machine learning model.

Abbreviations: ACA; anterior cerebral artery, ACoA; anterior communicating artery, BA; basilar artery, Fasudil; fasudil hydrochloride hydrate, ICA; internal carotid artery, IVR; interventional radiology, MCA; middle cerebral artery, mRS; modified Rankin Scale, OR; odds ratio, PCA; posterior cerebral artery, PICA; posterior inferior cerebellar artery, VA; vertebral artery, WFNS; World Federation of Neurosurgical Societies, *; $p < 0.05$, **; $p < 0.01$, ***; $p < 0.001$.

†; Presence of angiographical and symptomatic vasospasm has strong correlation ($r = 0.862$, $p < 0.001$ by Spearman's correlation coefficient). Therefore, to avoid multicollinearity, only symptomatic vasospasm was included in the multivariable analysis.
