# Supplementary material for: Machine Learning-Identified Potential Interaction Between Clazosentan and Nicardipine in Patients with Subarachnoid Hemorrhage
Source: J Clin Med. 2026 Feb 10;15(4):1383. doi: 10.3390/jcm15041383 (PMC12941893; doi:10.3390/jcm15041383)
Supplement: Supplementary file 1 [file jcm-15-01383-s001.zip › JCM_SupTable S9_20260119_mRS6m timevariable.pdf]

Supplementary Table S9. Multivariable logistic regression analysis of risk factors for poor outcomes (mRS score ≥ 3) at 6 months (sensitivity analysis) (n = 406)

	Univariable analysis			Multivariable analysis	
	mRS score 0–2 at 6 months (n = 269, 66.3%)	mRS score 3–6 at 6 months (n = 137, 33.7%)	p values	OR (95% confidence interval)	p values
Post-April 2022 (%)	161 (59.9%)	75 (54.7%)	0.324	1.10 (0.58–2.07)	0.760
Age; 60 ≤ years (%)	170 (63.2%)	118 (86.1%)	<0.001***	3.38 (1.86–6.14)	<0.001***
Sex (%female)	194 (72.1%)	99 (72.3%)	0.976	0.80 (0.45–1.46)	0.472
Past history					
Hypertension (%)	95 (35.3%)	63 (46.0%)	0.037*	1.72 (0.99–2.99)	0.054
Diabetes mellitus (%)	35 (13.0%)	16 (11.7%)	0.702		
Stroke (%)	5 (1.9%)	8 (5.8%)	0.032*	1.62 (0.39–6.69)	0.500
WFNS grade II–V compared to I as reference			0.349	0.77 (0.40–1.40)	0.350
Interaction between clazosentan and WFNS grade II–V				6.36 (1.89–21.42)	0.003**
Aneurysm size (mm), mean (SD)	5.5 (2.5)	6.9 (4.7)	<0.001***	1.14 (1.03–1.26)	0.011*
Interaction between clazosentan and aneurysm size				1.01 (0.82–1.23)	0.968
Aneurysm location			0.479		
ACA or ACoA (%)	78 (29.0%)	48 (35.0%)			
ICA (%)	100 (37.2%)	41 (29.9%)			
MCA (%)	63 (23.4%)	33 (24.1%)			
VA, BA, PCA, PICA (%)	28 (10.4%)	15 (11.0%)			
Fisher CT group					
1−3 (%)	237 (88.1%)	99 (72.3%)			
4 (%)	32 (11.9%)	38 (27.7%)	<0.001***	2.41 (1.20–4.84)	0.013*
Surgical procedure					
Endovascular coiling, not surgical clipping (%endovascular coiling)	131 (48.7%)	58 (42.3%)	0.224		
Spinal drainage (%)	125 (46.5%)	51 (37.2%)	0.076		
Ventricular drainage (%)	54 (20.1%)	58 (42.3%)	<0.001***	3.81 (1.95–7.45)	<0.001***
Interaction between clazosentan and ventricular drainage				1.67 (0.48–5.84)	0.478
Cisternal drainage (%)	49 (18.2%)	36 (26.3%)	0.059		
Cerebral vasospasm prophylaxis and other medications					

Clazosentan (%)	125 (46.5%)	34 (24.8%)	<0.001***	0.12 (0.02–0.75)	0.024*
Fasudil (%)	201 (74.7%)	116 (84.7%)	0.022*	0.59 (0.22–1.57)	0.293
Cilostazol (%)	194 (72.1%)	96 (70.1%)	0.666		
Statin (%)	116 (43.1%)	66 (48.2%)	0.330		
Nicardipine (%)	55 (20.4%)	25 (18.2%)	0.599		
Antiepileptic drug (%)	127 (47.2%)	54 (39.4%)	0.135		
Outcomes and complications					
Angiographic vasospasm (%)	41 (15.2%)	43 (31.4%)	<0.001***	†	
Symptomatic vasospasm (%)	24 (8.9%)	43 (31.4%)	<0.001***	2.61 (1.21–5.65)	0.015*
IVR against cerebral vasospasm (%)	12 (4.5%)	9 (6.6%)	0.355		
Cerebral infarction (%)	48 (17.8%)	54 (39.4%)	<0.001***	1.54 (0.76–3.15)	0.234
Cerebral complication (%)	49 (18.2%)	60 (43.8%)	<0.001***	3.18 (1.71–5.91)	<0.001***
Systemic complication (%)	71 (26.4%)	71 (51.8%)	<0.001***	2.33 (1.34–4.02)	0.003**

As a sensitivity analysis, we additionally included a binary calendar-time variable (post–April 2022 period) in the multivariable models to account for potential time-related practice changes. There were no missing values. In this multivariable analysis, we included the items that were significant in the univariable analysis, along with the three items with the largest SHapley Additive exPlanations interaction values in the machine learning model.

Abbreviations: ACA; anterior cerebral artery, ACoA; anterior communicating artery, BA; basilar artery, Fasudil; fasudil hydrochloride hydrate, ICA; internal carotid artery, IVR; interventional radiology, MCA; middle cerebral artery, mRS; modified Rankin Scale, OR; odds ratio, PCA; posterior cerebral artery, PICA; posterior inferior cerebellar artery, VA; vertebral artery, WFNS; World Federation of Neurosurgical Societies, *; $p < 0.05$, **; $p < 0.01$, ***; $p < 0.001$.

†; Presence of angiographic and symptomatic vasospasm has strong correlation ($r = 0.862$, $p < 0.001$ by Spearman's correlation coefficient), so only symptomatic vasospasm was included in the multivariable analysis considering the multicollinearity.
